# Genomic and Phenotypic Characterization of Experimentally Selected Resistant Leishmania donovani Reveals a Role for Dynamin-1-Like Protein in the Mechanism of Resistance to a Novel Antileishmanial Compound

**DOI:** 10.1128/mbio.03264-21

**Published:** 2022-01-11

**Authors:** Aya Hefnawy, Gabriel Negreira, Marlene Jara, James A. Cotton, Ilse Maes, Erika D’Haenens, Hideo Imamura, Bart Cuypers, Pieter Monsieurs, Christina Mouchtoglou, Hans De Winter, Isabel Pintelon, Jean-Pierre Timmermans, Matt Berriman, Mandy Sanders, Julio Martin, Geraldine de Muylder, Jean-Claude Dujardin, Yann G.-J. Sterckx, Malgorzata Anna Domagalska

**Affiliations:** a Molecular Parasitology Unit, Institute of Tropical Medicine, Antwerp, Belgium; b Wellcome Sanger Institute, Wellcome Genome Campus, Hinxton, United Kingdom; c Laboratory of Medicinal Chemistry, University of Antwerp, Antwerp, Belgium; d Department of Veterinary Sciences, University of Antwerp, Antwerp, Belgium; e Global Health R&D, GlaxoSmithKline, Tres Cantos, Madrid, Spain; f Department of Biomedical Sciences, University of Antwerp, Antwerp, Belgium; g Laboratory of Medical Biochemistry, University of Antwerp, Antwerp, Belgium; h Infla-Med Centre of Excellence, University of Antwerp, Antwerp, Belgium; University of Geneva

**Keywords:** CRISPR-Cas9, *Leishmania*, drug resistance mechanisms, dynamin, genomics

## Abstract

The implementation of prospective drug resistance (DR) studies in the research-and-development (R&D) pipeline is a common practice for many infectious diseases but not for neglected tropical diseases (NTDs). Here, we explored and demonstrated the importance of this approach using as paradigms Leishmania donovani, the etiological agent of visceral leishmaniasis (VL), and TCMDC-143345, a promising compound of the GlaxoSmithKline (GSK) “Leishbox” to treat VL. We experimentally selected resistance to TCMDC-143345 *in vitro* and characterized resistant parasites at the genomic and phenotypic levels. We found that it took more time to develop resistance to TCMDC-143345 than to other drugs in clinical use and that there was no cross-resistance to these drugs, suggesting a new and unique mechanism. By whole-genome sequencing, we found two mutations in the gene encoding the L. donovani dynamin-1-like protein (LdoDLP1) that were fixed at the highest drug pressure. Through phylogenetic analysis, we identified LdoDLP1 as a family member of the dynamin-related proteins, a group of proteins that impacts the shapes of biological membranes by mediating fusion and fission events, with a putative role in mitochondrial fission. We found that L. donovani lines genetically engineered to harbor the two identified LdoDLP1 mutations were resistant to TCMDC-143345 and displayed altered mitochondrial properties. By homology modeling, we showed how the two LdoDLP1 mutations may influence protein structure and function. Taken together, our data reveal a clear involvement of LdoDLP1 in the adaptation/reduced susceptibility of L. donovani to TCMDC-143345.

## INTRODUCTION

The life span of any antimicrobial drug is, unfortunately, limited; its clinical use generally represents a new step in the arms race between human creativity and pathogen adaptability. Sooner or later, drug resistance (DR) or another phenotypic adaptation arises (1). Human countermeasures (such as new therapeutic regimens or combination therapy) can be adopted, but the drug will ultimately have to be replaced by a new compound, if any are available. Understanding the process of DR and developing strategies to counter it are particularly critical for neglected tropical diseases (NTDs), for which there are typically only a few drugs in the therapeutic arsenal and the research-and-development (R&D) pipeline (2). It is essential to safeguard existing compounds and develop new ones. At the same time, studies on molecular mechanisms of resistance are classically applied to understand the mode of action of antimicrobial agents because of the possibility that the resistance determinants are caused by specific genetic variations resulting in altered target binding to the drug molecule.

For NTDs, DR is generally studied retrospectively once DR has already been established in clinical settings. In a recent opinion paper, we recommended to keep one step ahead in the arms race and implement prospective DR studies in the R&D pipeline, a common practice for many infectious diseases but not for NTDs. Two specific recommendations have been given so far (2): (i) exploiting resources of parasite biobanks to test the efficacy of novel compounds on a wider range of parasites, including recent isolates from clinically relevant settings for the prospective use of the compound, a practice shown to be highly relevant (3), and (ii) experimentally selecting DR to new lead compounds and characterizing it broadly to assess the adaptive skills of the parasite for the compound, guide further drug development, and help counter DR if it develops in clinical practice.

Here, using (i) Leishmania donovani (the etiological agent of visceral leishmaniasis [VL], which is fatal if left untreated) and (ii) TCMDC-143345, a promising compound of the GlaxoSmithKline (GSK) “Leishbox,” pan-active against Trypanosoma cruzi, Trypanosoma brucei, and L. donovani (4), including antimony-resistant isolates (3), and the chemical starting point of the CF series of DNDi (https://dndi.org/research-development/portfolio/cf-series/), as paradigms, we experimentally selected resistance to the compound and proceeded to a genomic and phenotypic characterization of DR parasites. We found that it took more time to develop resistance to TCMDC-143345 than to other drugs in clinical use and that there was no cross-resistance to these drugs, suggesting a new and unique mechanism. Whole-genome characterization of independent TCMDC-143345-resistant lines highlighted two mutations in the gene encoding L. donovani dynamin-1-like protein (LdoDLP1) that were fixed at the highest drug pressure. Through phylogenetic analysis, we identified LdoDLP1 as a family member of the dynamin-related proteins (DRPs), a group of proteins that impacts the shapes of biological membranes by mediating fusion and fission events, with a putative role in mitochondrial fission. We genetically engineered our L. donovani strain to harbor the two identified LdoDLP1 mutations: parasites were resistant to TCMDC-143345 and displayed altered mitochondrial properties. The results are further supported by homology modeling, which provides insights as to how the two LdoDLP1 mutations may influence protein structure and function. Taken together, the data presented in this paper reveal a clear involvement of LdoDLP1 in the adaptation/reduced susceptibility of L. donovani to TCMDC-143345. Our results also demonstrate the practical relevance of prospective drug resistance studies to guide the R&D pipeline and future clinical applications of this compound.

## RESULTS

### Experimental resistance of promastigotes to TCMDC-143345 takes 50 weeks to establish, is stable, and is maintained in amastigotes.

To assess the ability of parasites to adapt to TCMDC-143345 we set up an experimental resistance experiment in quadruplicate for TCMDC-143345 (lines A, B, C, and D), starting with 0.2 μM, and for comparison, we used miltefosine (MIL) pressure in duplicate (lines A and B), starting with 2 μM. For TCMDC-143345, two lines (A and B) were lost during the selection process after round 4. Selection was continued from round 5 with line C and lines D1 to D4, generated by splitting the original line D at this round (see Fig. S1 and Data Set S1A in the supplemental material).

10.1128/mbio.03264-21.2 FIG S1 Flowchart describing the TCMDC-143345 resistance selection process. Rounds at which DNA was extracted are named sets (1 to 7). Rounds 1 to 3 started with four replicates, A, B, C, and D (4 lines maintained with TCMDC-143345). In round 4 (lines A and B were lost due to contamination), line D was subcultured into 4 flasks (lines D1 to -4). Line C was difficult to maintain under high drug concentrations, which is why lower drug pressure was maintained. At round 7 and due to limited compound availability, only lines C, D1, and D3 were maintained. IC_50_ values in micromolar are shown in the white boxes. Genomes of all lines were sequenced unless otherwise mentioned (line lost or some intermediate steps). Download FIG S1, TIF file, 0.4 MB. Copyright © 2022 Hefnawy et al. 2022 Hefnawy et al. https://creativecommons.org/licenses/by/4.0/ This content is distributed under the terms of the Creative Commons Attribution 4.0 International license.

10.1128/mbio.03264-21.10 DATA SET S1 (A) Summary of all IC_50_ values (micromolar) observed in promastigotes for all compounds and in amastigotes for compound TCMDC-14345, as shown in Fig. S2 in the supplemental material. We also provide a summary of resistance selection rounds at which DNA extraction was undertaken, the corresponding selection pressure with TCMDC-143345, and IC_50_s. The WT and DMSO controls were maintained *in vitro* in parallel to drug resistance selection, without and with DMSO, respectively: DNA was extracted from both controls in all sets except set 4. (B) Table showing the alternative allele frequency values of the 245 identified SNPs. The values for resistant lines C and D1 to -4 as well as the WT and DMSO control lines are shown at different stages of resistance selection. (C) GO enrichment results obtained with g:Profiler for the entire chromosome 6 and chromosome 31 and segments of chromosomes 17 and 30. As g:Profiler currently supports only Leishmania major, orthology mappings from L. donovani to L. major are also displayed below the results. (D) Additional data on chemical features of TCMDC-143345. Download Data Set S1, XLSX file, 1.3 MB. Copyright © 2022 Hefnawy et al. 2022 Hefnawy et al. https://creativecommons.org/licenses/by/4.0/ This content is distributed under the terms of the Creative Commons Attribution 4.0 International license.

10.1128/mbio.03264-21.3 FIG S2 Stability of resistance in promastigotes and resistance phenotypes in amastigotes. (A) Susceptibilities of LdBPK_282 cl4 WT and TCMDC-143345-resistant line D1 promastigotes as well as line D1-no drug (maintained for 20 weeks without drug pressure) to TCMDC-143345. Averages from 4 independent experiments are shown. (B) Susceptibilities of LdBPK_282 cl4 WT and TCMDC-143345-resistant line D1 amastigotes in THP-1 cells to TCMDC-143345. The *x* axis shows the log_10_ values of the drug concentrations used (in micromolar). Averages from 3 independent experiments are shown. (C to F) Cross-resistance. Susceptibilities of LdBPK_282 cl4 WT and TCMDC-143345-resistant line D1 promastigotes as well as line D1-no drug (maintained for 20 weeks without drug pressure) to known compounds (Sb^III^ [C], MIL [D], and Ampho B [E]) as well as compound Y (DNDI-6690) (F) were determined. The *x* axis shows the log values of the drug concentrations used (in micromolar). Averages from 4 independent experiments are shown. (G and H) *In vitro* fitness in the absence of drug pressure. Growth curves of promastigotes of the LdBPK_282 cl4 WT and the TCMDC-143345-resistant lines D3 and C (G) and engineered mutants (H) together with their respective controls are shown. The inset shows a subset of the growth curves (day 0 to day 3). The points represent the means from three biological replicates, and shading represents the confidence interval for the smoothed locally estimated scatterplot smoothing (LOESS) curve. Download FIG S2, TIF file, 0.9 MB. Copyright © 2022 Hefnawy et al. 2022 Hefnawy et al. https://creativecommons.org/licenses/by/4.0/ This content is distributed under the terms of the Creative Commons Attribution 4.0 International license.

**FIG 1:**
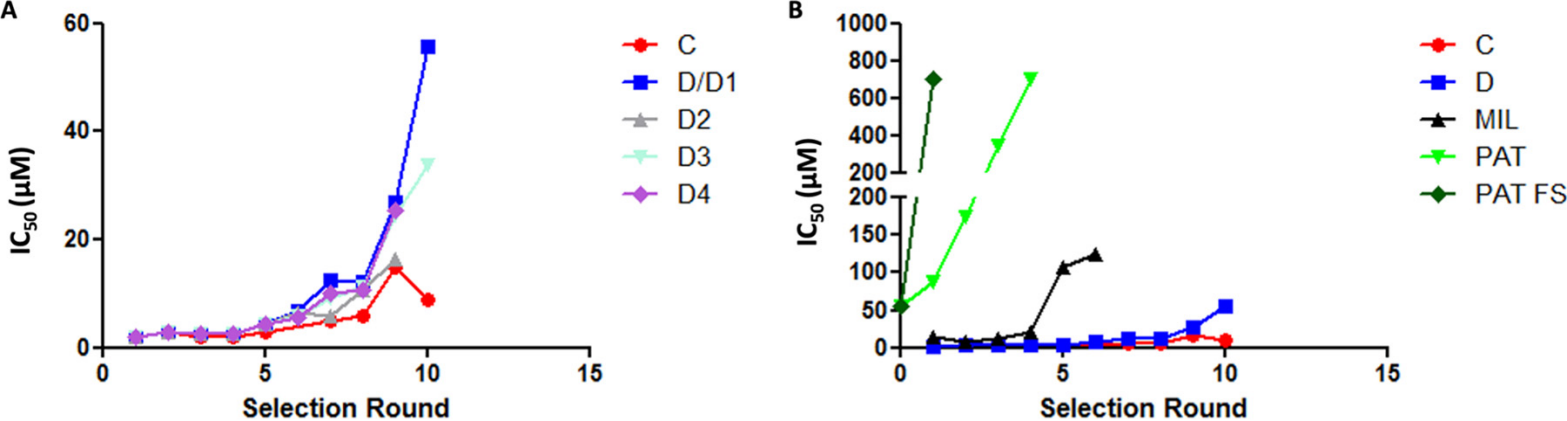
Time to resistance during selection experiments. The IC_50_ is shown in micromolar on the *y* axis, and the selection round is shown on the *x* axis (approximately 5 weeks per round), in the different lines of LdBPK_282 cl4 kept under pressure with TCMDC-143345 (A) and in lines C and D1 compared with LdBPK_282 cl4 selected for resistance to different known compounds (miltefosine [MIL] and potassium antimonyl tartrate [PAT]) (B). PAT FS, potassium antimonyl tartrate treatment consisting of “flash” selection exposing the parasites directly to the highest concentration of the drug (5).

The first parameter to be evaluated was the time to resistance (5), which was defined as the time needed for each line to display a wild-type (WT) growth curve in the presence of the highest selection pressure following the stepwise selection process. The selection dynamics for various lines are shown in Fig. 1A. It took 10 selection rounds (time to resistance of approximatively 50 weeks) for line D1 to reach the highest TCMDC-143345 50% inhibitory concentration (IC_50_) (55 μM). In comparison, the IC_50_ of TCMDC-143345 for the WT line maintained without drug pressure for the same time was 2 to 3 μM. The selection dynamics for line C were rather different from those for line D1, and resistance selection was not complete even after 10 rounds, thereby implying a longer time to resistance. At round 5 of selection, line C was not adapting well, which is why the pressure was decreased. The highest IC_50_ achieved was 15 μM at round 9. For the remaining line D replicates, line D3 had a profile of reduced susceptibility similar to that of line D1, while for lines D2 and D4, selection had to be stopped one round earlier (IC_50_ ranging between 16 and 25 μM) (Fig. S1) due to the limited availability of TCMDC-143345.

To place these results into context, the time to resistance for MIL was assessed in parallel and was about 30 weeks (6 selection rounds), with a shift of the IC_50_ from 13 μM to 100 μM. For comparison, we added data from our previous study with the same L. donovani MHOM/NP/03/BPK282/0 clone 4 (LdBPK_282 cl4) strain, in which we showed that the time to resistance to trivalent antimonials was 20 to 5 weeks (depending on the selection protocol) (5). Figure 1B shows clearly that the time to resistance was the longest for TCMDC-143345. The second evaluated parameter was the stability of the resistance phenotype. Both the WT line and line D1 were maintained for 20 weeks without drug pressure and then challenged again with TCMDC-143345. From Fig. S2A, it can be observed that the withdrawal of drug pressure for a prolonged period of time did not alter the susceptibility of line D1 to TCMDC-143345 (IC_50_ of >25 μM). Third, the susceptibility of intracellular amastigotes was assessed. TCMDC-143345 pressure was applied on THP-1 macrophages infected with line D1. Intracellular amastigotes of line D1 showed an IC_50_ of 30 μM versus 2 μM for the WT, confirming that the resistance selected at the promastigote stage was maintained at the amastigote stage (Fig. S2B). Fourth, promastigotes of the resistant line D1, the WT, and line D1-no drug (D1 line maintained for 20 weeks without drug pressure) were tested for their susceptibility to known antileishmanial compounds (MIL, amphotericin B [Ampho B], and Sb^III^) as well as one novel compound (DNDI-6690) that has a chemical structure closely related to that of TCMDC-143345. No cross-resistance was observed for Ampho B and Sb^III^, with similar IC_50_ values observed for all lines (Fig. S2C and E). There was increased susceptibility of the TCMDC-143345-resistant lines to MIL (Fig. S2D). Interestingly, all TCMDC-143345-resistant lines showed higher IC_50_ values for DNDI-6690 than the WT, thereby implying cross-resistance of the TCMDC-143345-resistant lines to DNDI-6690 (Fig. S2F). All IC_50_ values are shown in Data Set S1A. Finally, we looked at the *in vitro* fitness of the TCMDC-143345-resistant promastigotes in the absence of the drug. The resistant lines had a moderate but significantly lower rate of growth than the wild type (Fig. S2G and Text S1). Of all the resistant lines, line C displayed the slowest growth.

10.1128/mbio.03264-21.1 TEXT S1 (1) Methods. (1.1) Promastigotes and susceptibility tests; (1.2) mammalian cell cultures; (1.3) multiple-regression model for the evaluation of the effect of resistance and engineered mutations on parasite growth; (1.4) mapping of sequencing reads, SNP/indel prediction, and gene ontology; (1.5) somy of chromosomes; (1.6) CRISPR-Cas9-mediated engineering of *Leishmania*; (1.7) phylogenetic analyses; (1.8) transmission electron microscopy. (2) Results and discussion. (2.1) Growth curves of resistant and engineered lines; (2.2) single nucleotide polymorphism; (2.3) aneuploidy and local copy number variation; (2.4) CRISPR-Cas9-mediated introduction of point mutations into LdoDLP1; (2.5) homology modeling of LdoDLP1. Download Text S1, DOCX file, 0.05 MB. Copyright © 2022 Hefnawy et al. 2022 Hefnawy et al. https://creativecommons.org/licenses/by/4.0/ This content is distributed under the terms of the Creative Commons Attribution 4.0 International license.

### Missense mutations in the gene encoding the dynamin-1-like protein LdoDLP1 can be found in independently selected lines resistant to TCMDC-143345.

**FIG 2:**
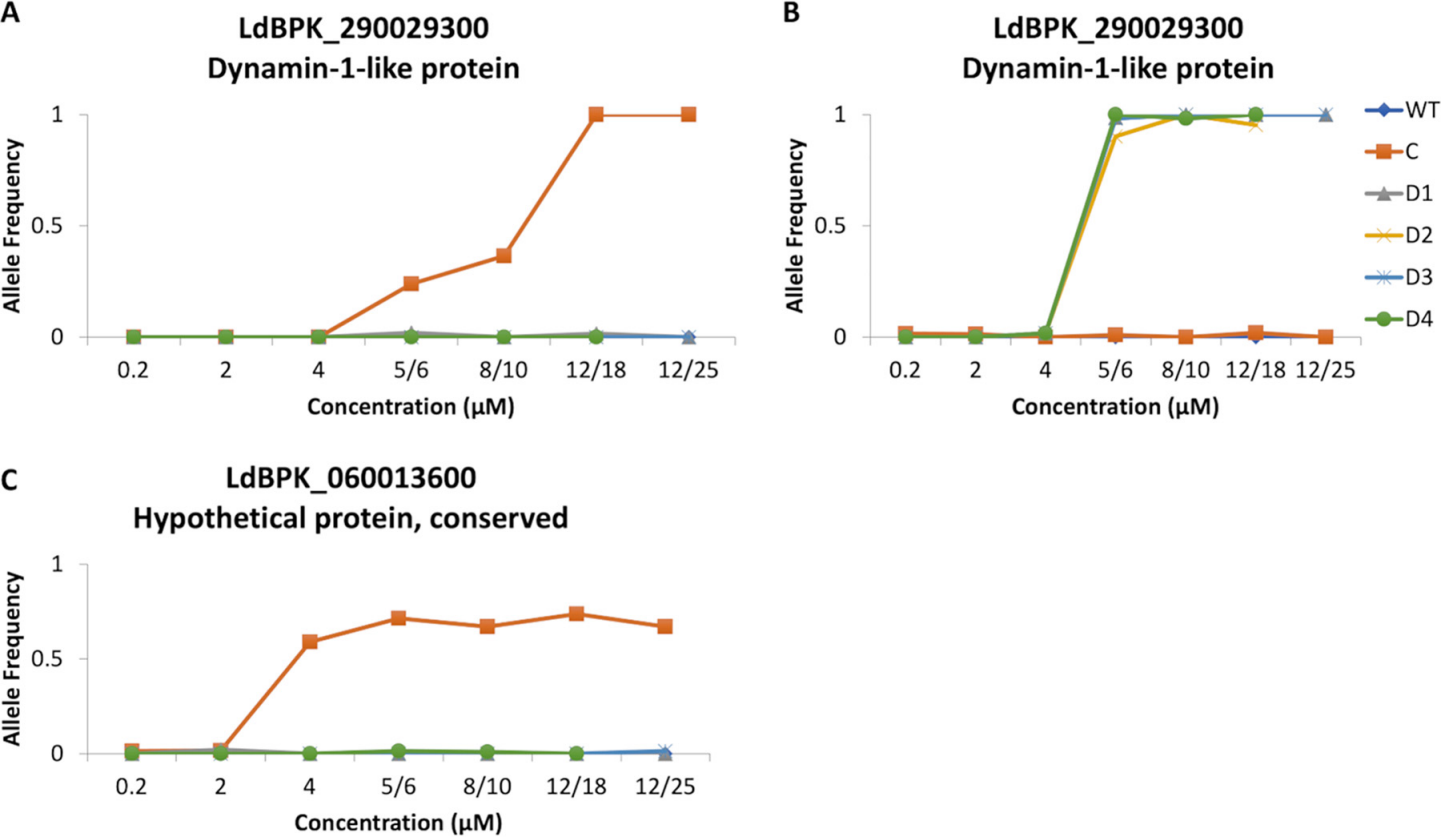
Major SNPs and their allele frequency changes observed during selection of TCMDC-143345 resistance. (A and B) C:T missense mutation at position Ld29:1000024 (A) and C:A missense mutation at position Ld29:999029C (B), both in the gene encoding dynamin-1-like protein (LdoDLP1) (LdBPK_290029300). (C) G:T missense mutation at position Ld06:353587 in a conserved hypothetical protein (LdBPK_060013600).

To identify genetic changes underlying the observed reduced susceptibility to TCMDC-143345, we applied whole-genome sequencing (WGS), and we characterized the genomic changes of nuclear DNA in the control WT and the resistant C and D1 lines at all steps of the selection process (Fig. S1). First, we analyzed changes at the level of nucleotide sequence, i.e., single nucleotide polymorphisms (SNPs) and small insertion and deletions (indels). A total of 245 SNP variants not present at the start of drug selection were identified in all the lines (Data Set S1B). Among these, only 2 missense mutations with large and statistically significant changes in allele frequency were observed in line C (Fig. 2A and C). The first one appeared at 5 μM TCMDC-143345, with a gradual increase in the allele frequency from 0 to 1 in LdBPK_290029300, the gene encoding dynamin-1-like protein (LdoDLP1). This C:T missense mutation translates to a change from alanine at position 324 to threonine (Ala324Thr). The second significant change in the allele frequency in line C concerns another missense mutation, G:T in chromosome 6 (chr6); the allele frequency shifts to 0.5 at 4 μM TCMDC-143345 in a conserved hypothetical protein (LdBPK_060013600) and stabilizes at around 0.7 during further selection. Only one missense mutation with a significant change in the allele frequency has been observed in all D lines (Fig. 2B). It starts at 6 μM TCMDC-143345, changes from 0 to 1, and stays stable until the end of the selection pressure. Intriguingly, this mutation is also located in the gene encoding LdoDLP1 (LdBPK_290029300) but results in a different change at the amino acid level compared to the LdoDLP1 mutation found in line C; i.e., glutamate at position 655 changes to aspartate (Glu655Asp). These results were supported by using a testing approach inspired by burden tests for rare disease associations, confirming that the number of mutations in this gene is most highly correlated with the IC_50_ (Text S1 and Fig. S3). No significant indels were detected in line C or D (D1 to -4) along the selection pressure.

10.1128/mbio.03264-21.4 FIG S3 Genomic changes during selection of resistance to TCMDC-143345. (A) Somy changes in lines D1 to D4 and the WT and DMSO control lines. The heat map represents the karyotype dynamics across the selection of resistance of LdBPK_282 cl4 to TCMDC-143345. The color key shows the normalized chromosome read depth (S). (B and C) Long copy number variation of the subtelomeric fragment. The phenomenon is observed in chr17 (B) and chr30 (C) during selection of resistance to TCMDC-143345. (D to G) Burden test for detecting significant SNPs during the selection experiment. Correlations between the total allele frequencies of nonreference alleles of a given gene and the IC_50_ values were determined. Plots are shown for the four genes with significant correlations when corrected for multiple testing. (D) Dynamin-1-like protein LdoDLP1 (LdBPK_290029300); (E) protein kinase (LdBPK_190009300); (F) MGT2 magnesium transporter (LdBPK_250016900); (G) mitochondrial RNA binding complex 1 subunit (LdBPK_310012800). Download FIG S3, GIF file, 0.7 MB. Copyright © 2022 Hefnawy et al. 2022 Hefnawy et al. https://creativecommons.org/licenses/by/4.0/ This content is distributed under the terms of the Creative Commons Attribution 4.0 International license.

**FIG 3:**
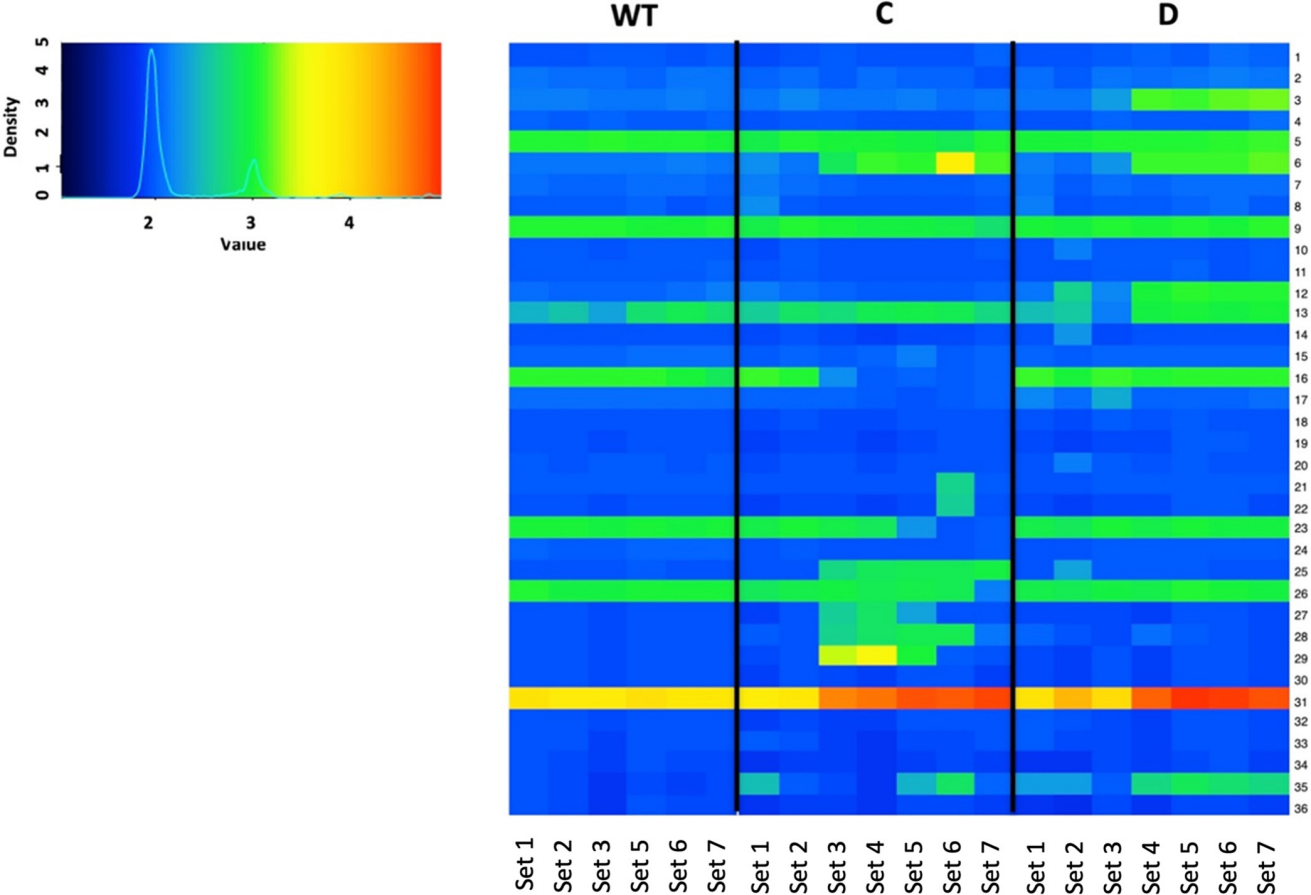
Somy changes in TCMDC-143345-resistant lines C and D1 and WT controls. The heat map represents the karyotype dynamics across the selection of resistance of LdBPK_282 cl4 to TCMDC-143345. The color key shows the normalized chromosome read depth (S). Sets 1 to 7 of the different lines are shown with the following added drug concentrations: 0.2 μM, 2 μM, 4 μM, 5 μM, 8 μM, 12 μM, and 12 μM for line C and 0.2 μM, 2 μM, 4 μM, 6 μM, 10 μM, 18 μM, and 25 μM for line D, respectively. WT parasites were sequenced at the same time points as for lines C and D, except for set 4 (hence, there are only 6 time points).

As we previously showed that aneuploidy and local copy number variations (CNVs) occur at an early stage of the selection process (6), we wondered whether this would be the case in this experiment. Aneuploidy was already present before selection, but additional aneuploid chromosomes were observed at around 4 to 6 μM TCMDC-143345, and final patterns of aneuploidy at the end of selection were rather different between lines C and D1 (Fig. 3). Few CNVs were observed, and the most striking were amplifications/deletions of large subtelomeric chromosomal stretches, also line specific, in chr17 (line D1) and chr30 (line C) (Fig. S3B and C). More details on aneuploidy and CNVs can be found in Text S1.

### The CRISPR-Cas9-mediated mutants and overexpression lines confirm the role of LdoDLP1 in resistance to TCMDC-143345.

**FIG 4:**
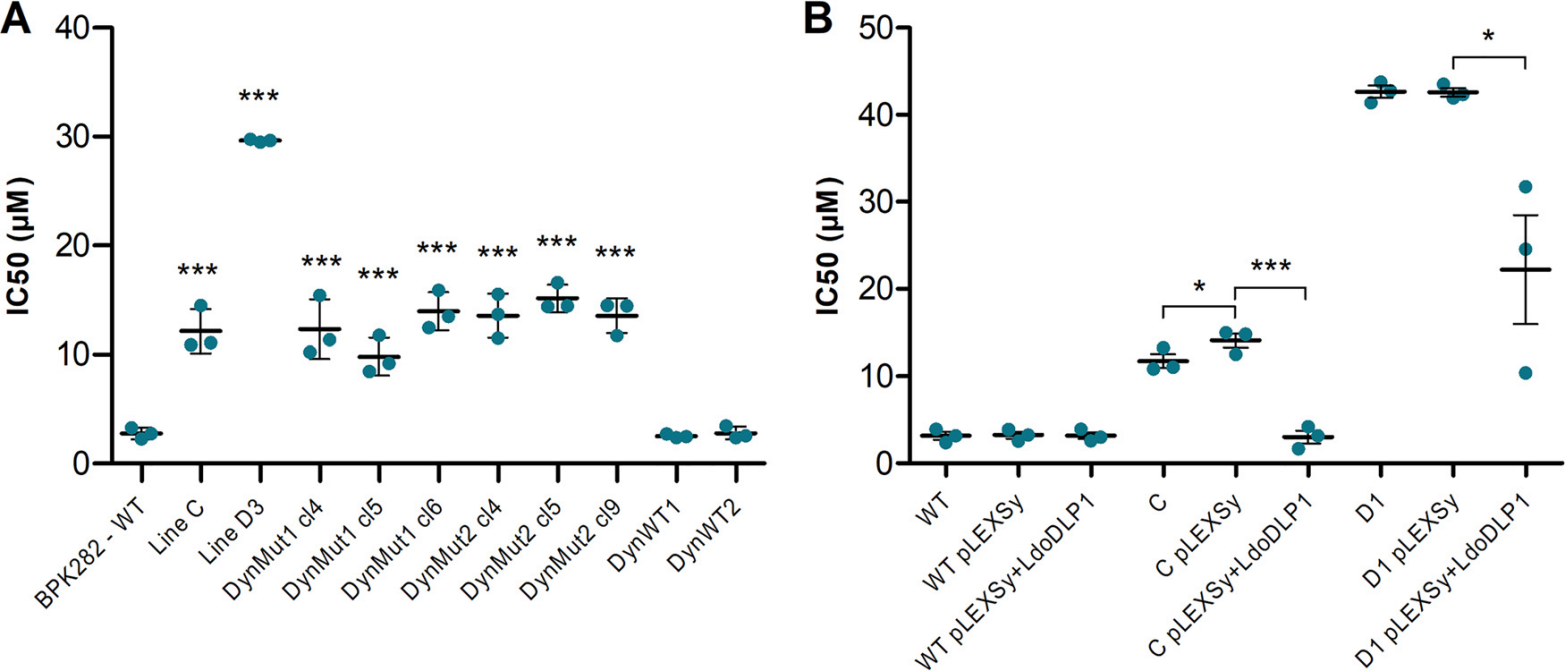
(A) Susceptibility of 3 clones with the Ala324Thr (DynMut1 cl4, cl5, and cl6) or the Glu655Asp (DynMut2 cl4, cl5, and cl9) LdoDLP1 mutation introduced by CRISPR-Cas9. WT LdBPK_282 cl4 and unselected parasites transfected with DynWT1 or DynWT2 sgRNA and DNA templates were included as controls. Lines C and D3 were included for comparison. (B) Susceptibly of lines C and D1 overexpressing the WT LdoDLP1 gene. Lines represent the averages and standard deviations of data from three independent replicates (dots) of each experiment. Each dot represents the average among 3 technical replicates. *, *P* < 0.05; ***, *P* ≤ 0.001 (by ANOVA with Bonferroni’s multiple-comparison test).

As the only common missense mutation in the two independent resistant lines was found in LdoDLP1, we hypothesized that the genetic variation in this gene is responsible for the reduced susceptibility to TCMDC-143345. To test this hypothesis, we recreated the identified mutations in wild-type promastigotes by means of a modified CRISPR-Cas9 system described previously (7), and we analyzed their susceptibility to TCMDC-143345. Detailed results for the selection of transfected promastigotes and subsequent controls are shown in Text S1. Three clones were derived from each of the selected lines (bearing the artificially introduced Ala324Thr or Glu655Asp mutations). The 6 clones were then submitted to a susceptibility test using a resazurin assay. The CRISPR-Cas9-engineered clones displayed a 4- to 5-fold increase in the IC_50_ of TCMDC-143345 compared to WT LdBPK_282 cl4 or the uncloned parasites from control transfections 2 and 7 with a donor DNA lacking the missense mutations (DynWT1 and DynWT2, respectively) (*P* < 0.001 by one-way analysis of variance [ANOVA]), achieving an IC_50_ similar to that for line C (Fig. 4A). Both the Ala324Thr (DynMut1 [mutation of line C]) and the Glu655Asp (DynMut2 [mutation of line D]) mutations had similar impacts on susceptibility to TCMDC-143345. Notably, no mutant clone displayed an IC_50_ similar to those observed in lines D. Growth curves in the absence of drug pressure showed similar growth rates between DynMut1 and DynMut2 and the pT007 control, the WT line, which constitutively expresses Cas9 protein (Fig. S2H and Text S1).

In parallel to the CRISPR-Cas9-induced mutagenesis experiment, we also investigated the effect of overexpressing the wild-type LdoDLP1 gene in lines C and D1. The transfection of an overexpression vector containing the wild-type form of the gene completely abrogated the reduced susceptibility to TCMDC-143345 in line C, reducing the IC_50_s to levels similar to those of wild-type LdBPK_282 cl4 (Fig. 4B) (*P* < 0.001 by one-way ANOVA). In line D1, however, while a significant reduction in the IC_50_ was observed (*P* < 0.001 by one-way ANOVA), parasites still demonstrated reduced susceptibility to the compound, with an average IC_50_ of 22 μM. Altogether, these results indicate that the mutations in LdoDLP1 constitute the major genetic changes responsible for the observed reduced susceptibility to TCMDC-143345 in line C, while in line D1, this is accompanied by additional mutations in LdoDLP1 (for instance, the heterozygous mutation at Ld29_999508 [see Data Set S1B, summary_SNPs&indels]) or elsewhere in the genome (for instance, the large CNV in chr17).

### Phylogenetic analysis suggests that LdoDLP1 plays a role in mitochondrial fission.

LdoDLP1 belongs to the family of dynamin-related proteins (DRPs), a group of proteins that impacts the shapes of biological membranes by mediating fusion and fission events. Given (i) the clear contributions of the Ala324Thr and Glu655Asp LdoDLP1 mutations to the TCMDC-143345 resistance phenotype and (ii) the absence of detailed biochemical studies on leishmanial DRPs, a phylogenetic analysis was performed to learn more about the protein’s possible biological function. As was reported previously by Morgan and colleagues in their work on Trypanosoma brucei DLP1 (8), a multiple-sequence alignment (MSA) followed by the construction of a rooted phylogenetic tree revealed that DRPs fall into different functional clades. Interestingly, LdoDLP1 clusters together with T. brucei DLP1 into the clade of DRPs involved in mitochondrial fission (Fig. S4), suggesting a role for LdoDLP1 in this biological process. It is noteworthy that MSAs reveal that LdoDLP1 residues Ala324 and Glu655 display 100% conservation among trypanosomatid DRPs. Compared to DRPs from various organisms (as performed to build the phylogenetic tree in Fig. S4), especially LdoDLP1 Glu655 displays high sequence identity (73.3%). These findings suggest that these residues play an important role in protein function.

10.1128/mbio.03264-21.5 FIG S4 Phylogenetic analysis of LdoDLP1. A representation of the rooted bootstrap consensus tree was constructed by PhyML based on the sequence alignment of the displayed sequences. The numbers above the branches indicate the bootstrap scores (the number of bootstrap samples out of the 1,000 runs that have the clade). The different DRP functional clades mentioned are color-coded as follows: yellow, mitochondrial fusion; blue, membrane dynamics of the outer chloroplast membrane; red, endocytosis and vesicle trafficking in animals; green, vesicle trafficking in plants; orange, mitochondrial fission; magenta, plate formation and cell division in plants. Download FIG S4, TIF file, 2.1 MB. Copyright © 2022 Hefnawy et al. 2022 Hefnawy et al. https://creativecommons.org/licenses/by/4.0/ This content is distributed under the terms of the Creative Commons Attribution 4.0 International license.

### TCMDC-143345-resistant lines exhibit lower mitochondrial membrane potential than susceptible, wild-type parasites.

**FIG 5:**
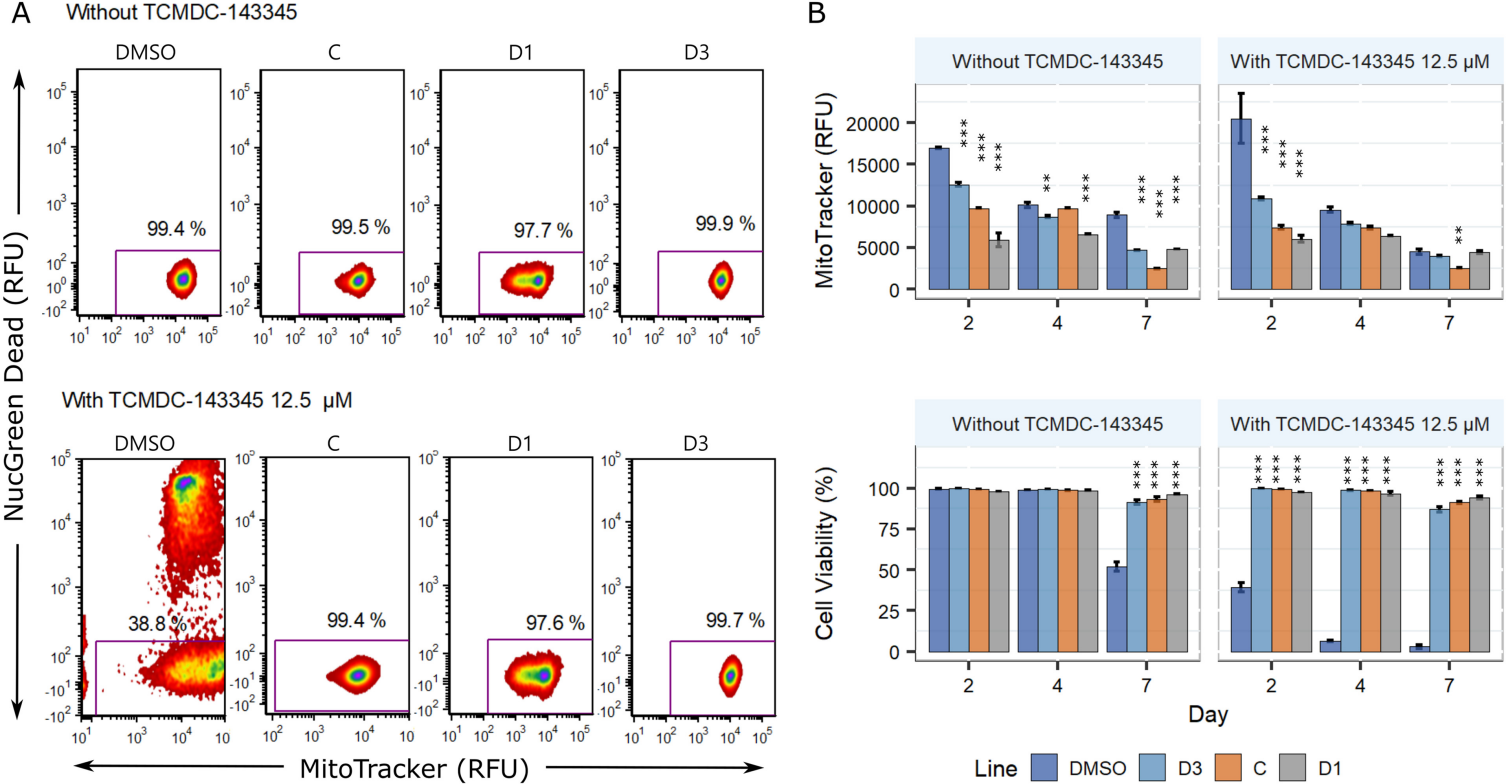
Mitochondrial membrane potential (MtMP) and cell viability of TCMDC-143345-resistant lines in promastigotes. (A) Density plots of MtMP as measured by the fluorescence of MitoTracker DeepRed and percent cell viability as measured by the fluorescence of NucGreen Dead. One plot of each line 2 days after treatment with 12.5 μM TCMDC-143345 and the respective controls are shown. (B) MtMP in WT (DMSO line) and resistant lines on days 2, 4, and 7 posttreatment. All the resistant lines had diminished MtMP in the logarithmic phase of culture (day 2) in comparison to the wild type in the presence or absence of TCMDC-143345. The differences between the wild-type and resistant lines were smaller at later days of incubation. Each bar represents the mean ± standard error of the mean (SEM) from three biological replicates. Within each day, the asterisks at the top of the bars represent significant differences between the resistant line and the wild type (*P* < 0.05 by Fisher’s LSD test).

It has been well established that mitochondrial dynamics play a role in the maintenance of normal mitochondrial membrane potential (MtMP) and cellular respiration (9, 10). Given the putative role of LdoDLP1 in mediating mitochondrial fission, we therefore evaluated whether the parasite lines containing LdoDLP1 mutations displayed changes in their mitochondrial activity. The MtMP and cell viability were simultaneously evaluated on logarithmic-phase (day 2), early-stationary-phase (day 4), and late-stationary-phase (day 7) promastigotes. In the presence or absence of TCMDC-143345, the overall trend was that cells with good viability in DR lines had decreased MtMP in comparison to the WT at days 2 and 4 (*P* < 0.001 by two-way repeated-measures [RM] ANOVA) (Fig. 5A and B). However, the differences between the WT and DR lines were larger in logarithmic-phase parasites (*P* values of <0.0001 by Fisher’s least significant difference [LSD] test) in both the presence and absence of TCMDC-143345. Noteworthy, also on day 2, the drug pressure altered the MitoTracker relative fluorescence units (RFU) dramatically in the WT but barely in the case of resistant lines (see scatterplots in Fig. 5A). Under TCMDC-143345 pressure, cell viability decreases significantly in the WT control, in comparison to the 3 resistant lines (Fig. 5B) (*P* < 0.001 for day and *P* < 0.001 for line by two-way ANOVA). Confocal images also indicate that mutants have a lower mitochondrial membrane potential; moreover, they show that the mitochondrial signal is more localized than in the WT. Since this may be an indicator of morphometric changes in the parasite’s single mitochondrion (Fig. S5A), we analyzed the parasites by transmission electron microscopy (TEM). Preliminary evaluation (Fig. S6) revealed some differences in the mitochondrial ultrastructures between mutant/resistant lines and the corresponding controls, such as enlargements of the mitochondrial section (DynMut1 cl4 and line D1) and crista size (heterogeneous, including some large cristae [500 nm] in DynMut1 cl4 and DynMut2 cl4). However, these preliminary observations should be completed by a more thorough analysis, which is beyond the scope of the present paper. We further evaluated if the CRISPR-engineered lines bearing the mutations Ala324Thr (DynMut1) and Glu655Asp (DynMut2) in LdoDLP1 also possessed diminished MtMP, and this was indeed confirmed for both mutations. Moreover, as with the *in vitro*-selected DR lines, the differences between LdoDLP1 mutant lines and the WT were larger during the logarithmic phase (Fig. S5B). Finally, we evaluated the effect of the overexpression of WT LdoDLP1 in resistant lines C and D1. MtMP increased in overexpressor C (adjusted *P* [*P*_adj_] < 0.001 by Tukey’s honestly significant difference [HSD] test), while there was no effect on line D1 (*P*_adj_ = 0.98 by Tukey’s HSD test). Altogether, these results indicate that for line C, the reduction in mitochondrial activity is mainly caused by the Ala324Thr mutation in LdoDLP1 and as such can be compensated for by overexpressing WT LdoDLP1. In line D1, the Glu655Asp mutation is also involved in the reduction of mitochondrial activity, but the additional genomic changes in this line (e.g., the trisomy of chromosomes 3 and 12) likely act in synergy, which is probably why full compensation cannot be achieved through the overexpression of WT LdoDLP1.

10.1128/mbio.03264-21.6 FIG S5 (A) Confocal microscopy for resistant lines and the WT after staining with MitoTracker. WT parasites had a strong signal coming from the anterior (distal part where the flagellum is protruding) until the posterior part of the body. This was expected as the single but large mitochondrion of *Leishmania* has a longitudinal localization. The mutant lines had dimmer signals but also a trend of more localized signals than the WT. Bars, 5 μm. (B) Mitochondrial membrane potential (MtMP) and cell viability in CRISPR-Cas9-engineered lines. The bars represent the results for the WT (DMSO line), pTB007, and LdoDLP1 mutants for Ala324Thr (DynMut1) and Glu655Asp (DynMut2) in the promastigote stage. pTB007 is the WT transfected with the CRISPR-Cas9 cassette to monitor the effect of the CRISPR-Cas9 engineering system. LdoDLP1 mutants had diminished MtMP in the logarithmic phase of culture (day 2) in comparison to the wild type in the presence or absence of TCMDC-143345. In the WT and pTB007, there were significant drug-induced alterations in the MtMP that were not seen in LdoDLP1 mutants. The single dynamin mutations conferred reduced susceptibility to TCMDC-143345 in both LdoDLP1 mutants as indicated by their high proportion of cells with good viability up to 4 days after exposure to the drug, which is in contrast to the very low cell viability of the WT and PTB007. Each bar represents the mean ± standard deviation (SD) from three biological replicates. The asterisks on top of the bars represent statistical significance between dichotomic comparisons after Tukey’s HSD test with the WT line as the reference. (C) MtMP for resistant lines overexpressing WT LdoDLP1. Only in resistant line C did the overexpression of LdoDLP1 reconstitute the MtMP to levels similar to those found in the WT. Asterisks represent statistical significance between dichotomic comparisons (resistant line versus its respective overexpressing line) after Tukey’s HSD test. Download FIG S5, TIF file, 1.7 MB. Copyright © 2022 Hefnawy et al. 2022 Hefnawy et al. https://creativecommons.org/licenses/by/4.0/ This content is distributed under the terms of the Creative Commons Attribution 4.0 International license.

10.1128/mbio.03264-21.7 FIG S6 Transmission electron microscopy (TEM) analysis of mitochondrial ultrastructures in different *Leishmania* strains. TEM of the mutant/resistant lines reveals some particular alterations in the ultrastructure of the mitochondria (m) compared to their corresponding controls. While the control groups (DMSO and pTB007) show distinct cross sections through the mitochondria of the parasites, including scattered cristae, mitochondria of the mutant/resistant lines often appear to be enlarged, as seen for the D1 and DynMut1 cl4 lines. In the DynMut1 cl4 and DynMut2 cl4 groups, cristae also tend to be longer and are often arranged in a more parallel pattern (arrowheads). Note that more vacuoles are present in the parasites of the DynMut2 cl4 group (asterisk) than in the control group or groups with other mutations. Bar, 500 nm. Download FIG S6, TIF file, 1.8 MB. Copyright © 2022 Hefnawy et al. 2022 Hefnawy et al. https://creativecommons.org/licenses/by/4.0/ This content is distributed under the terms of the Creative Commons Attribution 4.0 International license.

### Homology modeling suggests a molecular basis for the putative impact of the Ala324Thr and Glu655Asp mutations on LdoDLP1 function.

**FIG 6:**
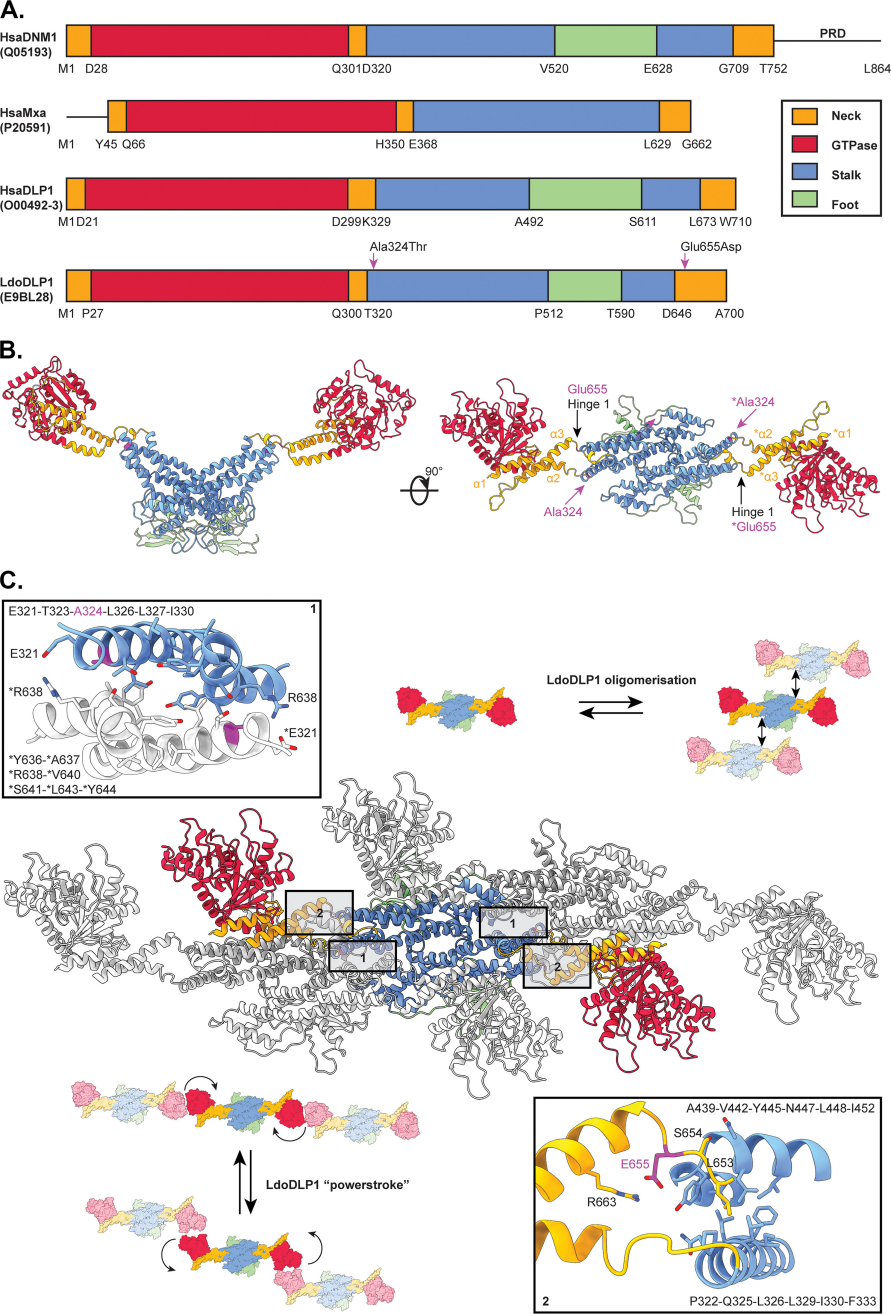
A homology model of LdoDLP1 suggests a molecular basis for the impact of TCMDC-143345 resistance mutations on protein function. (A) Schematic representation of the primary sequences of Homo sapiens dynamin-1 (HsaDNM1), H. sapiens myxovirus resistance protein 1 (HsaMxA), H. sapiens dynamin-like protein 1 (HsaDLP1), and LdoDLP1. The UniProt accession numbers are shown in parentheses. The different domains are color-coded, and their boundaries are indicated. The LdoDLP1 Ala324Thr and Glu655Asp mutations associated with reduced susceptibility to TCMDC-143345 are indicated by magenta arrows. (B) Cartoon representation of the LdoDLP1 dimer homology model, color-coded as in panel A. The 3 α-helices of the neck domain, hinge 1, and the positions of Ala324 and Glu655 are indicated for convenience. “*” indicates the second protomer of the LdoDLP1 dimer. (C) Model of a higher-order oligomer of LdoDLP1 (center). The central dimer is color-coded as in panel A, whereas the other two dimers are depicted in gray for clarity. The regions containing the Ala324Thr and Glu655Asp mutations are indicated by boxes “1” and “2,” respectively. The insets provide a closeup view of the molecular interactions in these boxed regions. Relevant amino acids are shown in a stick representation. Ala324 (in magenta) is proposed to be a part of the higher-order oligomerization interface of LdoDLP1 (box 1), while Glu655 (in magenta) is likely to play an important role in LdoDLP1’s powerstroke (box 2). “*” indicates the second protomer of the LdoDLP1 dimer.

Given that the introduction of the LdoDLP1 Ala324Thr and Glu655Asp point mutations bestows TCMDC-143345 resistance to the parasite and leads to an altered mitochondrial membrane potential, a structural model for LdoDLP1 was generated through homology modeling in an attempt to provide a molecular basis for these observations. LdoDLP1 contains all structural features characteristic of DRPs (Fig. 6A): a neck domain (also known as bundle-signaling element [BSE]) consisting of three α-helices, a GTPase domain, an α-helical stalk domain that contains the dimerization interface, and a foot domain (also known as the “paddle” or pleckstrin homology [PH] domain). In contrast to various other DRPs, LdoDLP1 is devoid of the intrinsically disordered proline-rich domain (PRD).

The Ala324Thr mutation maps onto a part of the stalk domain that is located close to the α2-helix of the neck domain (Fig. 6B) and is known to be responsible for the higher-order oligomerization of DRP dimers, which is important for DRP function in fission events (11–14) (for a more in-depth explanation, see Text S1). The Glu655Asp mutation is located in a region known as “hinge 1,” which connects the stalk domain with the α3-helix of the neck domain (Fig. 6B). Hinge 1 confers flexibility to DRPs, which is crucial for the so-called “hydrolysis-dependent powerstroke” underlying protein function (14–16) (for a more in-depth explanation, see Text S1). Hence, the LdoDLP1 mutations contributing to reduced susceptibility to TCMDC-143345 could (i) alter the tendency of LdoDLP1 dimers to form higher-order oligomers (Ala324Thr) and (ii) have a considerable impact on the flexibility of LdoDLP1’s hinge 1 region (Glu655Asp), which would in turn be expected to affect protein function.

## DISCUSSION

Humans and their pathogens are continuously locked in a molecular arms race, and human interventions like chemotherapy have a further impact on parasite adaptations and counteradaptations (1). This is well illustrated by the present study in which we (i) selected in L. donovani resistance to TCMDC-143345, a novel and potent antileishmanial compound (see chemical features in Data Set S1D in the supplemental material) that emerged from the Leishbox library of antileishmanial compounds (4), and (ii) characterized the extent of adaptations developed by the parasite.

Ten selection rounds (about 50 weeks) were necessary to obtain reduced susceptibility to TCMDC-143345 in promastigotes; resistance was stable in the absence of the drug, and it was also maintained in intracellular amastigotes. The same LdBPK_282 cl4 line was previously used for selecting and characterizing resistance to drugs used in clinical practice, thereby allowing comparisons of the development of resistance to different compounds. Overall, it took more time to develop resistance *in vitro* to TCMDC-143345 (10 rounds, 50 weeks) than to MIL (present study) (6 rounds, 30 weeks) and to Sb^III^ (1 to 4 selection rounds or 5 to 20 weeks, depending on the protocol used) (5).

Whole-genome sequencing is a powerful tool to identify molecular changes accompanying DR development in pathogens (5, 6, 17). In this study, in-depth genomic analysis revealed that TCMDC-143345-resistant lines were characterized by several aneuploidy changes: CNVs (see discussion in Text S1) and SNPs. Especially, the SNP analysis during the development of resistance turned out to be very informative. Out of 245 detected SNPs, only 2 missense mutations were fixed in 2 independent resistant lines, and both are located in the gene encoding L. donovani dynamin-1-like protein (LdoDLP1). We used CRISPR-Cas9 to recreate the Ala324Thr or the Glu655Asp mutations in the LdoDLP1 gene of WT LdBPK_282 cl4 promastigotes. Here, we demonstrated that these mutations independently confer reduced susceptibility to TCMDC-143345, leading to an IC_50_ for all mutant clones that is similar to that for line C. Consistent with this, the overexpression of the WT LdoDLP1 gene completely abolished the reduced susceptibility to TCMDC-143345 in line C, while it had only a partial impact on the IC_50_ of line D3. These observations demonstrate that loss-of-function mutations in the LdoDLP1 gene are sufficient to provide reduced susceptibility to TCMDC-143345 in both lines. However, in line D, this reduced susceptibility is further increased by other uncharacterized modifiers acting in concert with the mutation in LdoDLP1. We did not find any significant SNP or indel that might explain the higher reduced susceptibility of D lines, but they all share amplifications of chromosomes 3, 12, and 35. This is consistent with reports that aneuploidy in *Leishmania* is often associated with parasite drug resistance (18). Further work is needed to explore the functional impact of the specific aneuploidy changes observed in the D lines.

LdoDLP1 belongs to the family of dynamin-related proteins (DRPs), which are involved in several functions (8, 10): (i) mitochondrial fusion (fusion of the inner mitochondrial membrane), (ii) membrane dynamics of the outer chloroplast membrane, (iii) endocytosis and vesicle trafficking in animals, (iv) vesicle trafficking in plants, (v) mitochondrial fission (fission of the outer mitochondrial membrane), and (vi) plate formation and cell division in plants. DRPs cluster in different phylogenetic clades according to these functions (8), and our phylogenetic analysis showed a clear clustering of LdoDLP1 into the DRP clade involved in mitochondrial fission. The combined actions of mitochondrial fission and fusion govern mitochondrial dynamics, which underlie the organization, copy number, form, and function of mitochondria. The rates of these events are regulated according to the metabolic and/or developmental needs of the cell and in response to cellular stress or damage. While mitochondrial dynamics and cell division are not necessarily coupled in eukaryotes, the link between both processes seems to be very stringent in apicomplexan and kinetoplastid parasites. Both apicomplexans and kinetoplastids contain a single mitochondrion of which fission is controlled by a single or a limited number of DLPs (19, 20). For example, Toxoplasma gondii possesses three DLPs, and the ablation of one of these (TgDrpC) leads to drastic changes in mitochondrial morphology, growth defects, and, eventually, parasite death (20). Trypanosoma brucei harbors two DLP paralogs (TbDLP1 and TbDLP2) (21), of which especially TbDLP1 seems to play a central role in linking the processes of mitochondrial fission, cytokinesis, and the distribution of kinetoplastid DNA (8, 22–24). Interestingly, the abrogation of TbDLP1 function in T. brucei blocks mitochondrial fission and cell division, again leading to parasite fatality (8, 22). Similar to other protozoan parasites, *Leishmania* spp. contain a single elongated mitochondrion and harbor a single DLP (8).

The clear clustering of LdoDLP1 with T. brucei DLP1 into the DRP clade involved in mitochondrial fission hinted at a role for LdoDLP1 in mitochondrial dynamics, which is why we studied the mitochondrial membrane potential and cell viability of the TCMDC-143345-resistant parasite lines. In accordance with a proposed role of LdoDLP1 in mitochondrial fission, we found that all the TCMDC-143345-resistant lines (including the CRISPR-Cas9-generated LdoDLP1 Ala324Thr and Glu655Asp mutants) showed altered mitochondrial activity. Interestingly, homology modeling suggests that the LdoDLP1 Ala324Thr and Glu655Asp mutations associated with reduced susceptibility to TCMDC-143345 are located in the protein’s oligomerization interface and hinge 1 region, respectively, two regions that are essential for general DRP (and, thus, LdoDLP1) function. Hence, these mutations are likely to influence protein function, which in turn might explain the observed impact on mitochondrial dynamics within the TCMDC-143345-resistant L. donovani parasites.

The exact mechanism of action underlying the leishmanicidal effect of TCMDC-143345 and the precise role of LdoDLP1 in determining parasite reduced susceptibility remain unclear. In a first hypothesis, the LdoDLP1 mutations may be part of a compensation mechanism to alleviate the drug’s action on an as-yet-unidentified target (and its consequential detrimental effect on parasite viability). Within this context, it is interesting to note that leishmanial DLP was also proposed to be involved in the resistance profile of antimony- and miltefosine-resistant Leishmania infantum (25). In this proteomic study, L. infantum DLP was found to be downregulated in the antimony- and miltefosine-resistant strains, although no information was gathered with regard to possible mutations in the protein. In a second hypothesis, the drug exerts its deadly mode of action through LdoDLP1 binding (i.e., LdoDLP1 is the molecular target of TCMDC-143345), and the Ala324Thr and Glu655Asp mutations provide a direct escape mechanism. This hypothesis can equally be supported by the MtMP results. As of day 2 of drug exposure, we observed a drastic alteration in the MitoTracker RFU for the WT parasite, whereas the MitoTracker RFU remained unaffected for the resistant lines. This could be explained by the following scenarios: (i) the LdoDLP1 mutations in the DR lines prevent the compound from binding LdoDLP1 (i.e., the mutations are located in the binding site for TCMDC-143345), or (ii) they compensate for the effect that drug binding may have on LdoDLP1’s function in mitochondrial dynamics (TCMDC-143345 binds another LdoDLP1 ligand binding site). Clearly, in this hypothesis, the elucidation of the binding site and molecular interactions responsible for affinity and specific recognition between LdoDLP1 and TCMDC-143345 would reveal relevant insights to be exploited in the design of new compounds with optimized potency. This would be especially interesting since DLPs from protozoan parasites are considered drug targets because of their essentiality with regard to cell division and parasite growth (19). For the second hypothesis, it should be mentioned that targeting DLPs with small-molecule inhibitors to tackle disease through modulating mitochondrial dynamics is a known concept (26). Indeed, mammalian DRP1 (also clustering into the DRP clade involved in mitochondrial fission in our phylogenetic analysis) is actively being investigated as a target for treatment options for cancer and ischemia-reperfusion injury (27). Although the exact effect of DRP1 inhibitors is unclear, the following have been proposed: inhibition of DRP1 GTPase activity, causation of an increase in mitochondrial length, and/or modulation of reactive oxygen species (ROS) through an interaction with mitochondrial complex I (28). Whether LdoDLP1 is the molecular target of TCMDC-143345 (hypothesis 2) or is part of a coping mechanism without being the direct target of TCMDC-143345 (hypothesis 1) remains to be investigated.

Altogether, the results gathered in the present study demonstrate the practical relevance of prospective DR studies. The time to resistance shown here for TCMDC-143345 is encouraging in the context of the shelf life of this compound, but this should be complemented by *in vivo* studies. The demonstrated engagement of the unique leishmanial DLP in the resistance of L. donovani to TCMDC-143345 will allow the development of diagnostics targeting this gene to accompany further preclinical and clinical studies, if any, and it will also guide further investigation on the mode of action. The absence of cross-resistance with other drugs currently used in clinical practice qualifies TCMDC-143345 for future combination therapy if the compound would reach that stage. It is still early to assess whether this mechanism of resistance is relevant against other chemical classes, yet the resistant strains selected will become powerful tools to be employed with new chemical classes to ascertain whether they share biological space in terms of mode of action or resistance.

## MATERIALS AND METHODS

### Parasites.

We used L. donovani strain MHOM/NP/03/BPK282/0 clone 4 (called LdBPK_282 cl4 here) (for growth conditions, see Text S1 in the supplemental material), originally derived from a Nepalese patient with confirmed VL and cryopreserved at the Institute of Tropical Medicine in Antwerp, Belgium. The strain is considered sensitive to antimonials (Sb^III^), miltefosine (MIL), and amphotericin B (Ampho B), and it was used for determining the reference genome of L. donovani (29). The strain kept most of its intrinsic phenotypic features like virulence (29), transmissibility to sand flies (30), and natural drug susceptibility (5), thus constituting a good model for “real-life” parasites that will be exposed to new drugs under natural conditions.

### Selection of resistance and stability of resistance.

For the selection of resistance to TCMDC-143345, promastigotes were initially grown in quadruplicates (lines A, B, C, and D), and later on, because of the loss of lines A and B, line D was divided in 4, constituting a total of 5 lines (C and D1 to D4). MIL was used as a positive control for the experimental setup of drug resistance selection, and two duplicates were used (A and B). As negative controls, two additional lines were used: (i) the wild-type (WT) line LdBPK_282 cl4 maintained during the same passage numbers as the resistant lines but without drug pressure, and (ii) a WT line maintained with dimethyl sulfoxide (DMSO) (which was used as a solvent for TCMDC-143345). The resistant lines were maintained in the continuous presence of drugs, as described previously (5). Increasing concentrations of drugs were added in a stepwise manner until all lines grew at rates similar to those of wild-type parasites: (i) 0, 2, 10, 15, 60, and 100 μM for MIL and (ii) 0, 0.2, 1, 2, 4, 5, 6, 8, 12, and 12 μM for line C and 0, 0.2, 1, 2, 4, 5, 6, 10, 18, and 25 μM for line D for TCMDC-143345. Each selection round was approximately 5 weeks (2 passages per week), with the IC_50_ measured after each round. The selection flowchart is summarized in Fig. S1. To test the stability of the TCMDC-143345-resistant phenotype, the resistant line D1 was maintained for 20 weeks without drug pressure, after which the IC_50_ was measured.

### Promastigote susceptibility tests.

Susceptibility tests were performed after each selection round (drug resistance selection) or after parasite engineering (CRISPR-Cas9 or overexpression) (see below). The IC_50_s were determined on logarithmic-stage promastigotes after 72 h of exposure to TCMDC-143345 or MIL with a resazurin assay as previously described (6) and summarized in Text S1. For the cross-resistance experiments, the same protocol was used. The following maximal concentrations were used for testing the compounds: 50 μM for TCMDC-143345 and compound Y, 400 μM for MIL, 2 mM for Sb^III^, and 200 μM for Ampho B. Ten points of 1:2 dilutions were used per compound. Four independent experiments were run with technical duplicates per experiment.

### Amastigote susceptibility tests.

Phorbol myristate acetate (PMA; Sigma) (30 nM) was added to THP-1 cells (human monocytic leukemia; ATCC TIB-202) (for maintenance conditions, see Text S1) at 37°C for 48 h to differentiate these into adherent macrophages. Cells were washed and incubated with complete RPMI 1640 medium containing stationary-phase (day 6) L. donovani promastigotes at a macrophage/promastigote ratio of 1/30. After 5 h of incubation at 37°C, noninternalized promastigotes were removed by 3 successive washes with phosphate-buffered saline (PBS), and infected macrophages were incubated with TCMDC-143345 in RPMI 1640 medium supplemented with 5% heat-inactivated horse serum for 96 h. TCMDC-143345 was tested with a starting concentration of 25 μM in a 3-fold serial dilution. A 3-fold serial dilution of 3 μM amphotericin B was used as a positive control. Experiments were done in triplicate with technical duplicates per experiment. For confocal microscopy, infected cells were washed with PBS, fixed for 30 min with 4% formaldehyde, rinsed again with PBS, and stained with 4′,6′-diamidino-2-phenylindole (DAPI) (300 nM). Images were acquired with a Zeiss LSM 700 confocal microscope. The number of infected macrophages and the number of amastigotes per infected macrophage were determined by manual counting. These numbers obtained from the averages from counted wells were used to establish the infection index (percent infected macrophages × amastigotes/infected macrophages). IC_50_s were calculated with GraphPad Prism using a sigmoidal dose-response model with a variable slope.

### DNA and library preparation for whole-genome sequencing.

At each round of resistance selection (5 weeks of culture), parasites were harvested from lines C and D and from the two WT controls (maintained without and with DMSO). The list of samples sequenced is summarized in Data Set S1A. DNA isolation was done using a QIAamp DNA blood minikit (Qiagen), and the DNA concentration was assessed with the Qubit DNA broad-range DNA quantification kit (Thermo Fisher). Library preparation and sequencing of the different lines of the stepwise selection were performed at The Wellcome Sanger Institute (Hinxton, United Kingdom). Genomic DNA (gDNA) was sheared into 400- to 600-bp fragments by focused ultrasonication (Covaris adaptive focused acoustics technology; AFA Inc., Woburn, MA, USA). Amplification-free indexed Illumina libraries were prepared (31) using the NEBNext Ultra II DNA library prep kit (New England BioLabs). The libraries were quantified using the Accuclear ultrahigh-sensitivity double-stranded DNA (dsDNA) quantitative kit (Biotium) and then pooled in equimolar amounts. Paired-end reads of 150 bp were generated on the Illumina HiSeq X10 platform according to the manufacturer’s standard sequencing protocol (32).

### Whole-genome sequencing data analysis.

Somy, single nucleotide polymorphisms (SNPs), local copy number variations (CNVs), and indels were determined as described previously (29, 33) using the BPK282v2 PacBio reference genome (29); more details can be found in Text S1. SNPs and small indels were considered significantly different between parasite lines when the allele frequency showed a difference of at least 0.25 and a Mann-Whitney U test *P* value of <0.05 (34). Allele frequency shifts of >0.80 were considered homozygous variants. We used one criterion to evaluate whether a gene or chromosome copy number difference was biologically meaningful and statistically significant: the absolute difference in gene/chromosome copy number should be at least 0.5 to be significant. Gene ontology (GO) analyses were done as explained in Text S1. Heat maps were created using the heatmap3 package in R (R Development Core Team, 2015). Reasoning that mutations away from the sensitive parental strain at causative loci were likely to contribute to reduced susceptibility but that multiple variants in the same loci could contribute to the resistance phenotype, we adopted a simple approach to identify significant loci informed by burden tests used in rare-variant association studies (35). We summed the nonreference allele frequencies of variants at each locus for each sequence sample and tested for association by regression of these total allele frequencies per locus against the measured IC_50_ for that sample.

### CRISPR-Cas9-mediated engineering of *Leishmania*.

LdBPK_282 cl4 parasites were transfected with the linearized pTB007 vector, obtained from Eva Gluenz (University of Oxford, UK) (7). Transgenic parasites were selected with 25 μg/mL hygromycin starting 24 h after transfection, and a clone was isolated using a microdrop method (36). To introduce the Ala324Thr or the Glu655Asp mutation using the CRISPR-Cas9 system, the single guide RNAs (sgRNAs) DynMut1-gRNA and DynMut2-gRNA were designed targeting the sequences CAGCAGCTGTGCAGTGGGCT and GGCACTGCTCTCCGAGCCCCC, respectively (sites of mutation are underlined). The sgRNA templates for *in vivo* transcription were generated by PCR as previously described (7). The sequences of all primers used in this work are provided in Table S1. For each mutation, a double-stranded donor DNA bearing the missense mutation was generated by annealing synthetic oligonucleotides. The DNA repair templates also included synonymous nucleotide substitutions to distinguish the CRISPR-Cas9-mediated mutations from potential naturally occurring mutations. The Ala324Thr and Glu655Asp mutations were independently recreated by transfecting the respective sgRNA templates and donor dsDNAs using basic parasite Nucleofector kit 1 (Lonza) with the U-033 program according to the manufacturer’s recommendations. Control transfections were made by either transfecting each donor dsDNA without the respective sgRNA templates or replacing each donor dsDNA with the DynWT1 or DynWT2 dsDNAs, which lack the missense mutation. At 24 h posttransfection, 10^6^ parasites from each transfection were transferred to a 24-well plate in a final volume of 1 mL/well of hemoflagellate modified minimal essential medium (HOMEM) with 20% fetal bovine serum and 9 μM TCMDC-143345 or 0.1% DMSO (control). Plates were incubated at 26°C for 12 days, and cell viability was determined by flow cytometry using the NucRed Dead 647 ReadyProbes and Vybrant DyeCycle green dyes (Thermo Fisher). Parasites that survived and grew in the presence of TCMDC-143345 were transferred to culture flasks and kept under pressure with 6 μM TCMDC-143345 for 2 passages, when clones were isolated with a microdrop method (36) and grown in the absence of drug pressure.

10.1128/mbio.03264-21.9 TABLE S1 List of primers and oligonucleotides used for CRISPR-Cas9-mediated engineering. Download Table S1, DOCX file, 0.02 MB. Copyright © 2022 Hefnawy et al. 2022 Hefnawy et al. https://creativecommons.org/licenses/by/4.0/ This content is distributed under the terms of the Creative Commons Attribution 4.0 International license.

### Overexpression of the WT LdoDLP1 gene in lines C and D1.

The wild-type LdoDLP1 gene was PCR amplified from the gDNA of LdBPK_282 cl4 with the primers InF-LdDNM1-F and InF-LdDNM1-R and cloned into the NotI and NcoI sites of the pLEXSy-Hyg2.1 expression vector (Jena Bioscience). The plasmid was linearized with the SwaI enzyme and transfected into parasites of fully resistant lines C and D1 as well as the standard LdBPK_282 cl4 line using basic parasite Nucleofector kit 1 (Lonza) with the U-033 program. The empty, linearized pLEXSy-Hyg2.1 vector was also transfected into each line as a control. Parasites were selected and maintained with 50 μg/mL hygromycin after 24 h posttransfection.

### Phylogenetic analysis.

The amino acid sequences of several DRPs (for details, see Text S1) were aligned using MAFFT (37) to generate a sequence alignment from which a rooted phylogenetic tree was constructed through the maximum likelihood method using PhyML (38). Escherichia coli CrfC, which shares features with the DRP family members, was employed as an outgroup to root the phylogenetic tree. The reliability of the tree was verified by performing 1,000 bootstrap replicates.

### Mitochondrial membrane potential and cell viability.

(i) Selected resistant lines C, D1, and D3 together with the DMSO control, with the same number of passages; (ii) CRISPR-engineered mutants together with the WT line and the WT transfected with CRISPR-Cas9 and called pT007 (both used as controls); as well as (iii) lines overexpressing the wild-type LdoDLP1 gene were cultivated at a density of 1 × 10^6^ parasites without or with 12.5 μM TCMDC-143345. On days 2, 4, and 7, the mitochondrial membrane potential (MtMP) and cell viability were coevaluated with MitoTracker DeepRed and NucGreen, respectively (Thermo Fisher Scientific). Briefly, 1 volume of parasites was incubated with 2 volumes of a medium containing 0.1 μM cell tracker DeepRed and 1 drop/mL of NucGreen. The samples were incubated for 15 min at 26°C and subsequently repelleted by centrifugation at 1,500 × *g* for 5 min. The cells were resuspended in new medium and analyzed by flow cytometry (BD FACS Verse) in the medium-flow-rate mode. An unstained sample was included in each experiment as a negative control for the establishment of the autofluorescence and the gates for the selection of the populations positive and negative for both fluorochromes.

### Homology modeling.

A homology model for the LdoDLP1 dimer was generated using MODELLER (39) with the following crystal structures as the templates: Homo sapiens DLP1 (UniProt accession number O00429; PDB accession number 4BEJ) (40), Rattus norvegicus DNM1 (UniProt accession number P21575; PDB accession number 3ZVR) (41), H. sapiens DNM3 (UniProt accession number Q9UQ16; PDB accession number 5A3F) (11), and H. sapiens DNM1 (UniProt accession number Q05193; PDB accession number 3SNH) (42). The homology model has normalized discrete optimized protein energy (zDOPE) and genetic algorithm 341 (GA341) scores of −0.255 and 1.000, respectively, thereby indicating its reliability. Molecular graphics visualization and analysis were performed with UCSF ChimeraX (43).

### Data availability.

Raw data were deposited in the European Nucleotide Archive (ENA) with the accession numbers ERS441806 to ERS441816.

10.1128/mbio.03264-21.8 FIG S7 Characterization of the mutant lines generated by CRISPR-Cas9. (A) Scheme representing the design of the DNA repair templates used to artificially introduce the Ala324Thr (top) or the Glu655Asp (bottom) mutation with CRISPR-Cas9. The black bar represents the WT LdoDLP1 gene. The regions encompassed by the DNA repair templates are represented by a gray box. A part of the sequence of the DNA repair templates is given, together with the translated amino acid sequence. Synonymous mutations are represented in blue, while missense mutations are indicated in red. The sgRNA targets and the protospacer-adjacent motif (PAM) are highlighted. (B) Percentage of live cells in the transfected LdBPK_282 cl4 lines after 12 days under selection with 9 μM TCMDC-143345. The table indicates the combination of sgRNAs and DNA repair templates transfected in each group. (C) Sanger sequencing of the mutation sites in the LdoDLP1 genes of DynMut1 and DynMut2 cl4. The missense mutations (homozygous in all clones) are indicated by an asterisk. The heterozygous synonymous mutations are represented by S (C or G). The CTCCTC allele is a restriction site for the BseRI nuclease in the DynMutN1 clones (black box). The missense mutation together with the synonymous mutations in the DynMutN2 clones create a BamHI site (black box). (D) Confirmation of heterozygosity in the synonymous mutations in the DynMutN1 clones and homozygosity in the missense mutations in the DynMutN2 clones by digestion. The flanking region of each mutation site was PCR amplified and submitted to digestion with BseRI for mutation 1 (top) or BamHI for mutation 2 (bottom). For each clone, the undigested (u) and digested (d) PCR products are shown. Download FIG S7, GIF file, 0.7 MB. Copyright © 2022 Hefnawy et al. 2022 Hefnawy et al. https://creativecommons.org/licenses/by/4.0/ This content is distributed under the terms of the Creative Commons Attribution 4.0 International license.

## References

[1] Caljon G, De Muylder G, Durnez L, Jennes W, Vanaerschot M, Dujardin J-C. 2016. Alice in microbes’ land: adaptations and counter-adaptations of vector-borne parasitic protozoa and their hosts. FEMS Microbiol Rev 40:664–685. doi:10.1093/femsre/fuw018. 27400870

[2] Hefnawy A, Berg M, Dujardin JC, De Muylder G. 2017. Exploiting knowledge on Leishmania drug resistance to support the quest for new drugs. Trends Parasitol 33:162–174. doi:10.1016/j.pt.2016.11.003. 27993477

[3] Hefnawy A, Cantizani J, Peña I, Manzano P, Rijal S, Dujardin JC, De Muylder G, Martin J. 2018. Importance of secondary screening with clinical isolates for anti-leishmania drug discovery. Sci Rep 8:11765. doi:10.1038/s41598-018-30040-5. 30082744 PMC6078976

[4] Peña I, Pilar Manzano M, Cantizani J, Kessler A, Alonso-Padilla J, Bardera AI, Alvarez E, Colmenarejo G, Cotillo I, Roquero I, de Dios-Anton F, Barroso V, Rodriguez A, Gray DW, Navarro M, Kumar V, Sherstnev A, Drewry DH, Brown JR, Fiandor JM, Julio Martin J. 2015. New compound sets identified from high throughput phenotypic screening against three kinetoplastid parasites: an open resource. Sci Rep 5:8771. doi:10.1038/srep08771. 25740547 PMC4350103

[5] Dumetz F, Cuypers B, Imamura H, Zander D, D’Haenens E, Maes I, Domagalska MA, Clos J, Dujardin J-C, De Muylder G. 2018. Molecular preadaptation to antimony resistance in *Leishmania donovani* on the Indian subcontinent. mSphere 3:e00548-17. doi:10.1128/mSphere.00548-17. PMC5907651 29669889

[6] Shaw CD, Lonchamp J, Downing T, Imamura H, Freeman TM, Cotton JA, Sanders M, Blackburn G, Dujardin JC, Rijal S, Khanal B, Illingworth CJR, Coombs GH, Carter KC. 2016. In vitro selection of miltefosine resistance in promastigotes of Leishmania donovani from Nepal: genomic and metabolomic characterization. Mol Microbiol 99:1134–1148. doi:10.1111/mmi.13291. 26713880 PMC4832254

[7] Beneke T, Madden R, Makin L, Valli J, Sunter J, Gluenz E. 2017. A CRISPR Cas9 high-throughput genome editing toolkit for kinetoplastids. R Soc Open Sci 4:170095. doi:10.1098/rsos.170095. 28573017 PMC5451818

[8] Morgan GW, Goulding D, Field MC. 2004. The single dynamin-like protein of Trypanosoma brucei regulates mitochondrial division and is not required for endocytosis. J Biol Chem 279:10692–10701. doi:10.1074/jbc.M312178200. 14670954

[9] Chen H, Chomyn A, Chan DC. 2005. Disruption of fusion results in mitochondrial heterogeneity and dysfunction. J Biol Chem 280:26185–26192. doi:10.1074/jbc.M503062200. 15899901

[10] Giacomello M, Pyakurel A, Glytsou C, Scorrano L. 2020. The cell biology of mitochondrial membrane dynamics. Nat Rev Mol Cell Biol 21:204–224. doi:10.1038/s41580-020-0210-7. 32071438

[11] Reubold TF, Faelber K, Plattner N, Posor Y, Ketel K, Curth U, Schlegel J, Anand R, Manstein DJ, Noé F, Haucke V, Daumke O, Eschenburg S. 2015. Crystal structure of the dynamin tetramer. Nature 525:404–408. doi:10.1038/nature14880. 26302298

[12] Kalia R, Wang RYR, Yusuf A, Thomas PV, Agard DA, Shaw JM, Frost A. 2018. Structural basis of mitochondrial receptor binding and constriction by DRP1. Nature 558:401–405. doi:10.1038/s41586-018-0211-2. 29899447 PMC6120343

[13] Faelber K, Dietrich L, Noel JK, Wollweber F, Pfitzner AK, Mühleip A, Sánchez R, Kudryashev M, Chiaruttini N, Lilie H, Schlegel J, Rosenbaum E, Hessenberger M, Matthaeus C, Kunz S, von der Malsburg A, Noé F, Roux A, van der Laan M, Kühlbrandt W, Daumke O. 2019. Structure and assembly of the mitochondrial membrane remodelling GTPase Mgm1. Nature 571:429–433. doi:10.1038/s41586-019-1372-3. 31292547 PMC7116848

[14] Gao S, Von der Malsburg A, Dick A, Faelber K, Schröder GF, Haller O, Kochs G, Daumke O. 2011. Structure of myxovirus resistance protein A reveals intra- and intermolecular domain interactions required for the antiviral function. Immunity 35:514–525. doi:10.1016/j.immuni.2011.07.012. 21962493

[15] Chen Y, Zhang L, Graf L, Yu B, Liu Y, Kochs G, Zhao Y, Gao S. 2017. Conformational dynamics of dynamin-like MxA revealed by single-molecule FRET. Nat Commun 8:15744. doi:10.1038/ncomms15744. 28548099 PMC5458555

[16] Chappie JS, Mears JA, Fang S, Leonard M, Schmid SL, Milligan RA, Hinshaw JE, Dyda F. 2011. A pseudoatomic model of the dynamin polymer identifies a hydrolysis-dependent powerstroke. Cell 147:209–222. doi:10.1016/j.cell.2011.09.003. 21962517 PMC3185303

[17] Shaw CD, Imamura H, Downing T, Blackburn G, Westrop GD, Cotton JA, Berriman M, Sanders M, Rijal S, Coombs GH, Dujardin JC, Carter KC. 2020. Genomic and metabolomic polymorphism among experimentally selected paromomycin-resistant Leishmania donovani strains. Antimicrob Agents Chemother 64:e00904-19. doi:10.1128/AAC.00904-19. 31658971 PMC7187574

[18] Mannaert A, Downing T, Imamura H, Dujardin JC. 2012. Adaptive mechanisms in pathogens: universal aneuploidy in Leishmania. Trends Parasitol 28:370–376. doi:10.1016/j.pt.2012.06.003. 22789456

[19] Voleman L, Doležal P. 2019. Mitochondrial dynamics in parasitic protists. PLoS Pathog 15:e1008008. doi:10.1371/journal.ppat.1008008. 31751405 PMC6871780

[20] Melatti C, Pieperhoff M, Lemgruber L, Pohl E, Sheiner L, Meissner M. 2019. A unique dynamin-related protein is essential for mitochondrial fission in Toxoplasma gondii. PLoS Pathog 15:e1007512. doi:10.1371/journal.ppat.1007512. 30947298 PMC6448817

[21] Benz C, Stříbrná E, Hashimi H, Lukeš J. 2017. Dynamin-like proteins in Trypanosoma brucei: a division of labour between two paralogs? PLoS One 12:e0177200. doi:10.1371/journal.pone.0177200. 28481934 PMC5421789

[22] Chanez AL, Hehl AB, Engstler M, Schneider A. 2006. Ablation of the single dynamin of T. brucei blocks mitochondrial fission and endocytosis and leads to a precise cytokinesis arrest. J Cell Sci 119:2968–2974. doi:10.1242/jcs.03023. 16787942

[23] Jakob M, Hoffmann A, Amodeo S, Peitsch C, Zuber B, Ochsenreiter T. 2016. Mitochondrial growth during the cell cycle of Trypanosoma brucei bloodstream forms. Sci Rep 6:36565. doi:10.1038/srep36565. 27874016 PMC5118809

[24] Schneider A, Ochsenreiter T. 2018. Failure is not an option—mitochondrial genome segregation in trypanosomes. J Cell Sci 131:jcs221820. doi:10.1242/jcs.221820. 30224426

[25] Vincent IM, Racine G, Légaré D, Ouellette M. 2015. Mitochondrial proteomics of antimony and miltefosine resistant Leishmania infantum. Proteomes 3:328–346. doi:10.3390/proteomes3040328. 28248274 PMC5217391

[26] Webb M, Sideris DP, Biddle M. 2019. Modulation of mitochondrial dysfunction for treatment of disease. Bioorg Med Chem Lett 29:1270–1277. doi:10.1016/j.bmcl.2019.03.041. 30954429

[27] Lima AR, Santos L, Correia M, Soares P, Sobrinho-Simões M, Melo M, Máximo V. 2018. Dynamin-related protein 1 at the crossroads of cancer. Genes (Basel) 9:115. doi:10.3390/genes9020115. PMC5852611 29466320

[28] Mallat A, Uchiyama LF, Lewis SC, Fredenburg RA, Terada Y, Ji N, Nunnari J, Tseng CC. 2018. Discovery and characterization of selective small molecule inhibitors of the mammalian mitochondrial division dynamin, DRP1. Biochem Biophys Res Commun 499:556–562. doi:10.1016/j.bbrc.2018.03.189. 29601815 PMC6626631

[29] Dumetz F, Imamura H, Sanders M, Seblova V, Myskova J, Pescher P, Vanaerschot M, Meehan CJ, Cuypers B, De Muylder G, Späth GF, Bussotti G, Vermeesch JR, Berriman M, Cotton JA, Volf P, Dujardin JC, Domagalska MA. 2017. Modulation of aneuploidy in *Leishmania in vitro* and *in vivo* environments and its impact on gene expression. mBio 8:e00599-17. doi:10.1128/mBio.00599-17. 28536289 PMC5442457

[30] Seblova V, Dujardin J-C, Rijal S, Domagalska M, Volf P. 2019. ISC1, a new Leishmania donovani population emerging in the Indian sub-continent: vector competence of Phlebotomus argentipes. Infect Genet Evol 76:104073. doi:10.1016/j.meegid.2019.104073. 31629887

[31] Kozarewa I, Ning Z, Quail MA, Sanders MJ, Berriman M, Turner DJ. 2009. Amplification-free Illumina sequencing—library preparation facilitates improved mapping and assembly of (G+C)-biased genomes. Nat Methods 6:291–295. doi:10.1038/nmeth.1311. 19287394 PMC2664327

[32] Bronner IF, Quail MA, Turner DJ, Swerdlow H. 2014. Improved protocols for Illumina sequencing. Curr Protoc Hum Genet 80:18.2.1–18.2.42. doi:10.1002/0471142905.hg1802s80. 26270174

[33] Imamura H, Downing T, van den Broeck F, Sanders MJ, Rijal S, Sundar S, Mannaert A, Vanaerschot M, Berg M, de Muylder G, Dumetz F, Cuypers B, Maes I, Domagalska M, Decuypere S, Rai K, Uranw S, Bhattarai NR, Khanal B, Prajapati VK, Sharma S, Stark O, Schönian G, de Koning HP, Settimo L, Vanhollebeke B, Roy S, Ostyn B, Boelaert M, Maes L, Berriman M, Dujardin JC, Cotton JA. 2016. Evolutionary genomics of epidemic visceral leishmaniasis in the Indian subcontinent. Elife 5:e12613. doi:10.7554/eLife.12613. 27003289 PMC4811772

[34] Tihon E, Imamura H, Dujardin J-C, Van Den Abbeele J, Van den Broeck F. 2017. Discovery and genomic analyses of hybridization between divergent lineages of Trypanosoma congolense, causative agent of animal African trypanosomiasis. Mol Ecol 26:6524–6538. doi:10.1111/mec.14271. 28752916

[35] Lee S, Abecasis GR, Boehnke M, Lin X. 2014. Rare-variant association analysis: study designs and statistical tests. Am J Hum Genet 95:5–23. doi:10.1016/j.ajhg.2014.06.009. 24995866 PMC4085641

[36] Van Meirvenne N, Janssens PG, Magnus E. 1975. Antigenic variation in syringe passaged populations of Trypanosoma (Trypanozoon) brucei. 1. Rationalization of the experimental approach. Ann Soc Belg Med Trop 55:1–23. 1231658

[37] Katoh K, Misawa K, Kuma KI, Miyata T. 2002. MAFFT: a novel method for rapid multiple sequence alignment based on fast Fourier transform. Nucleic Acids Res 30:3059–3066. doi:10.1093/nar/gkf436. 12136088 PMC135756

[38] Guindon S, Dufayard J-F, Lefort V, Anisimova M, Hordijk W, Gascuel O. 2010. New algorithms and methods to estimate maximum-likelihood phylogenies: assessing the performance of PhyML 3.0. Syst Biol 59:307–321. doi:10.1093/sysbio/syq010. 20525638

[39] Sali A, Blundell T. 1993. Comparative modelling by satisfaction of spatial restraints. J Mol Biol 234:779–815. doi:10.1006/jmbi.1993.1626. 8254673

[40] Fröhlich C, Grabiger S, Schwefel D, Faelber K, Rosenbaum E, Mears J, Rocks O, Daumke O. 2013. Structural insights into oligomerization and mitochondrial remodelling of dynamin 1-like protein. EMBO J 32:1280–1292. doi:10.1038/emboj.2013.74. 23584531 PMC3642683

[41] Ford MGJ, Jenni S, Nunnari J. 2011. The crystal structure of dynamin. Nature 477:561–566. doi:10.1038/nature10441. 21927001 PMC4075756

[42] Faelber K, Posor Y, Gao S, Held M, Roske Y, Schulze D, Haucke V, Noé F, Daumke O. 2011. Crystal structure of nucleotide-free dynamin. Nature 477:556–562. doi:10.1038/nature10369. 21927000

[43] Goddard TD, Huang CC, Meng EC, Pettersen EF, Couch GS, Morris JH, Ferrin TE. 2018. UCSF ChimeraX: meeting modern challenges in visualization and analysis. Protein Sci 27:14–25. doi:10.1002/pro.3235. 28710774 PMC5734306

