# Supplementary figures and images for: Genomic and Phenotypic Characterization of Experimentally Selected Resistant Leishmania donovani Reveals a Role for Dynamin-1-Like Protein in the Mechanism of Resistance to a Novel Antileishmanial Compound
Source: mBio. 2022 Jan 11;13(1):e03264-21. doi: 10.1128/mbio.03264-21 (PMC8749414; doi:10.1128/mbio.03264-21)

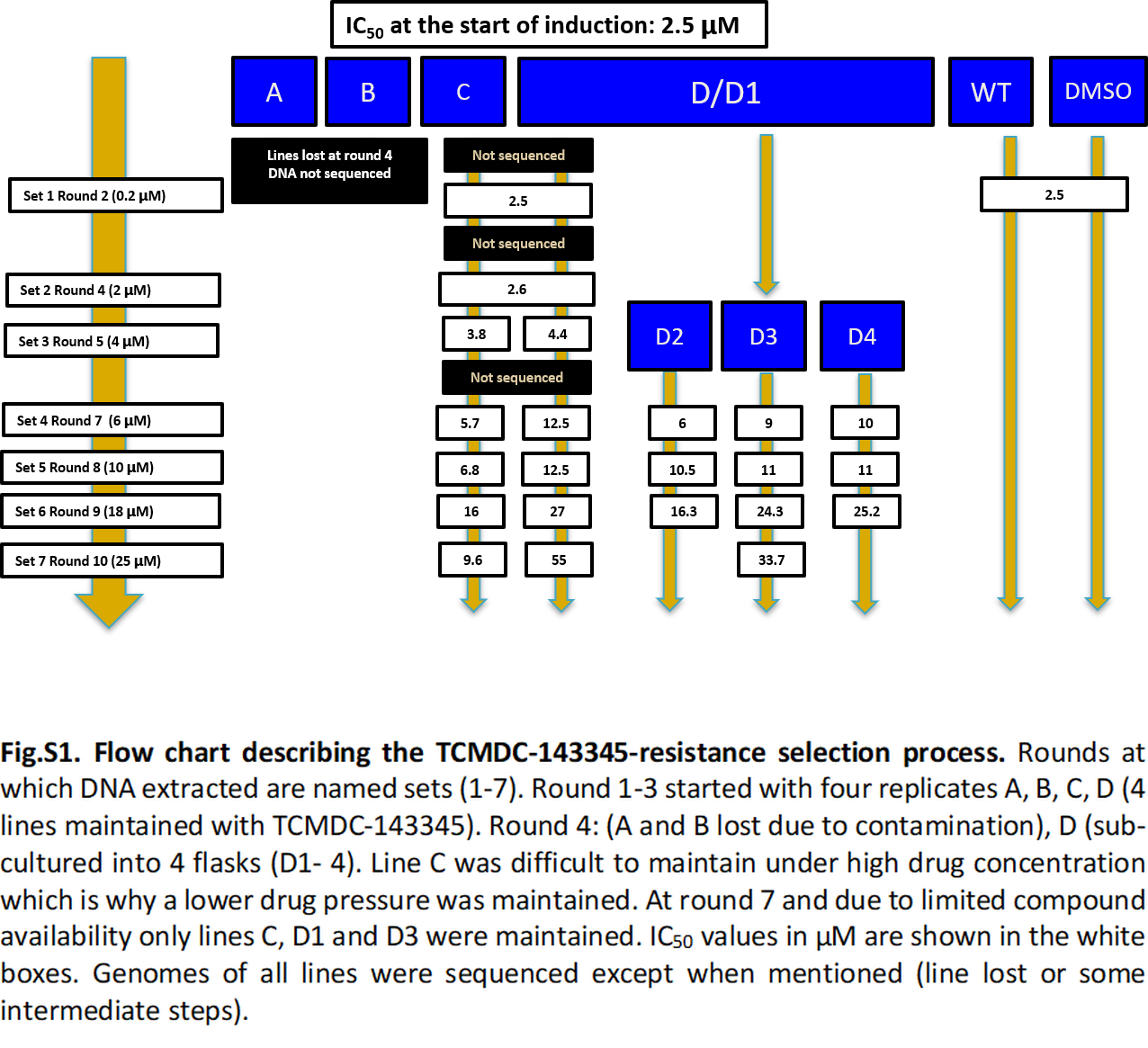

Supplement: FIG S1 [file mbio.03264-21-sf001.tif]

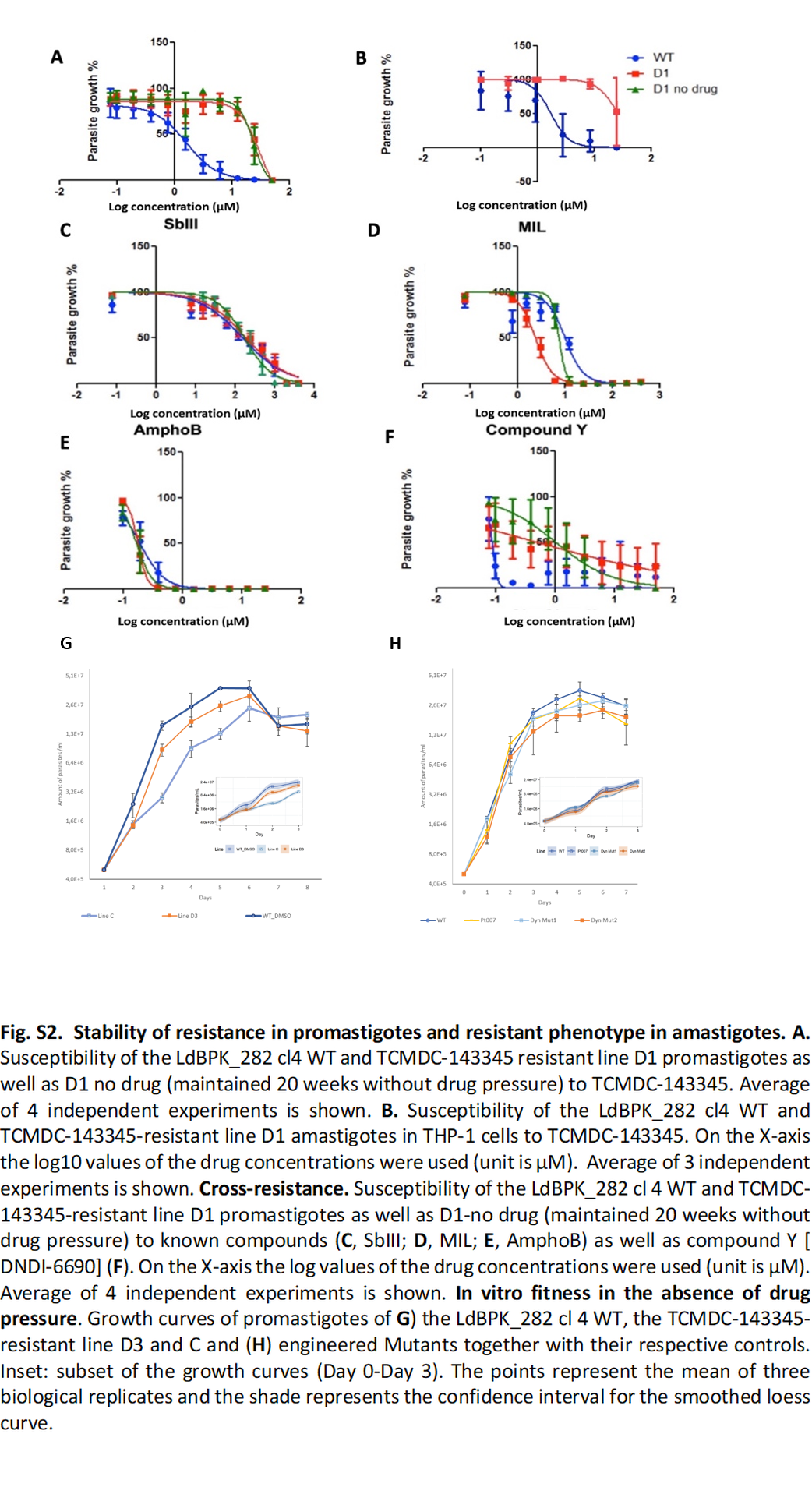

Supplement: FIG S2 [file mbio.03264-21-sf002.tif]

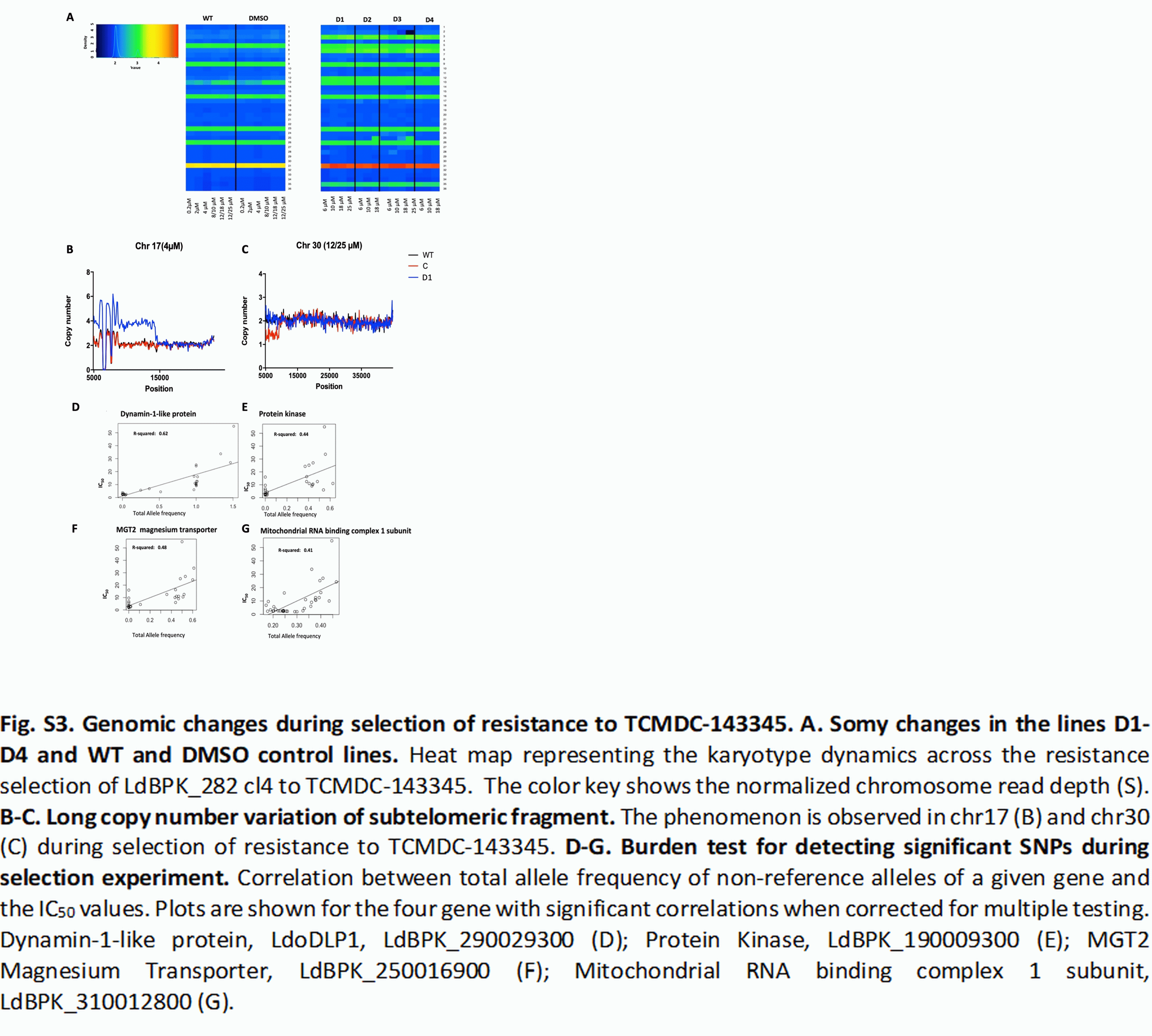

Supplement: FIG S3 [file mbio.03264-21-sf003.gif]

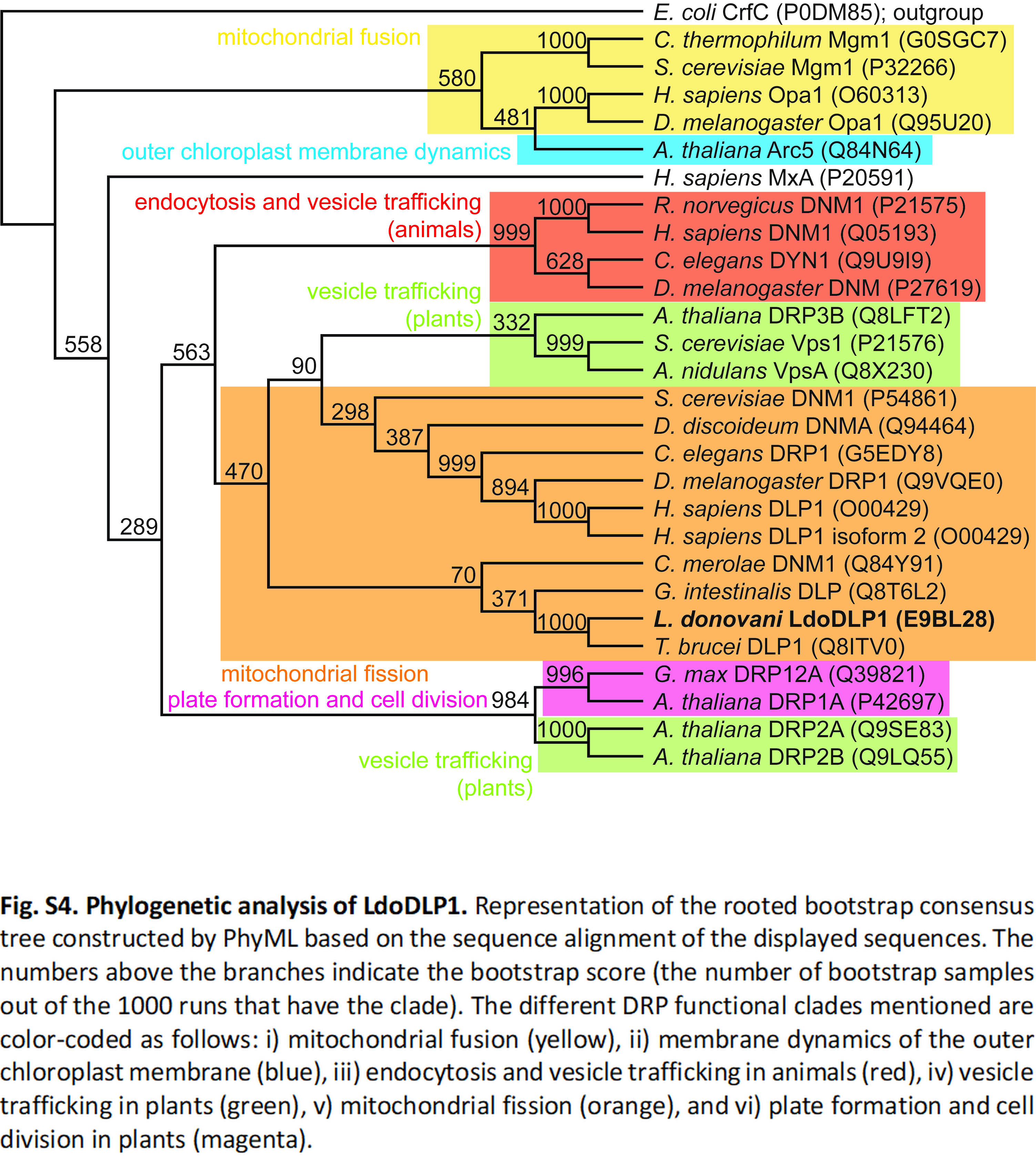

Supplement: FIG S4 [file mbio.03264-21-sf004.tif]

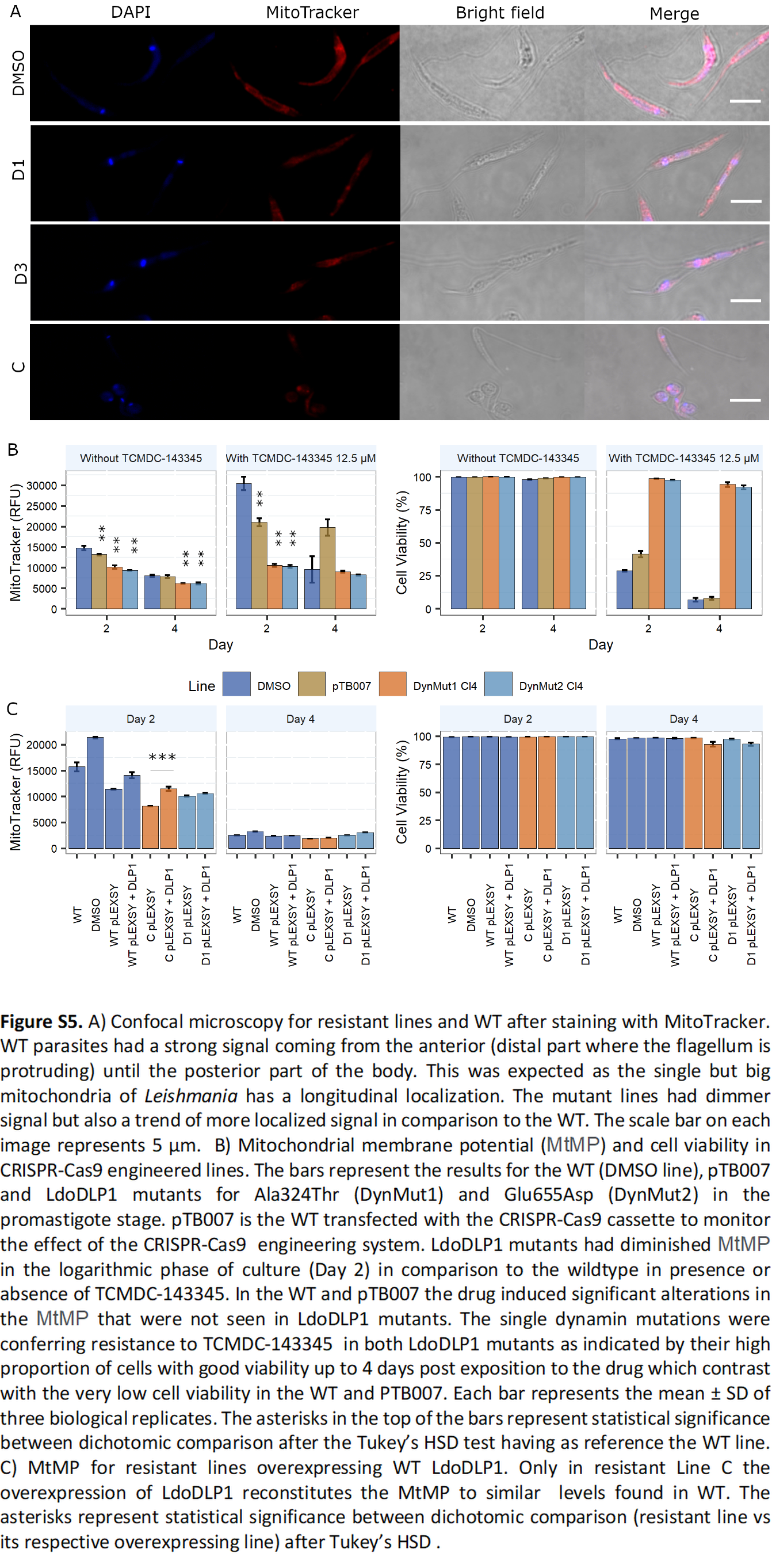

Supplement: FIG S5 [file mbio.03264-21-sf005.tif]

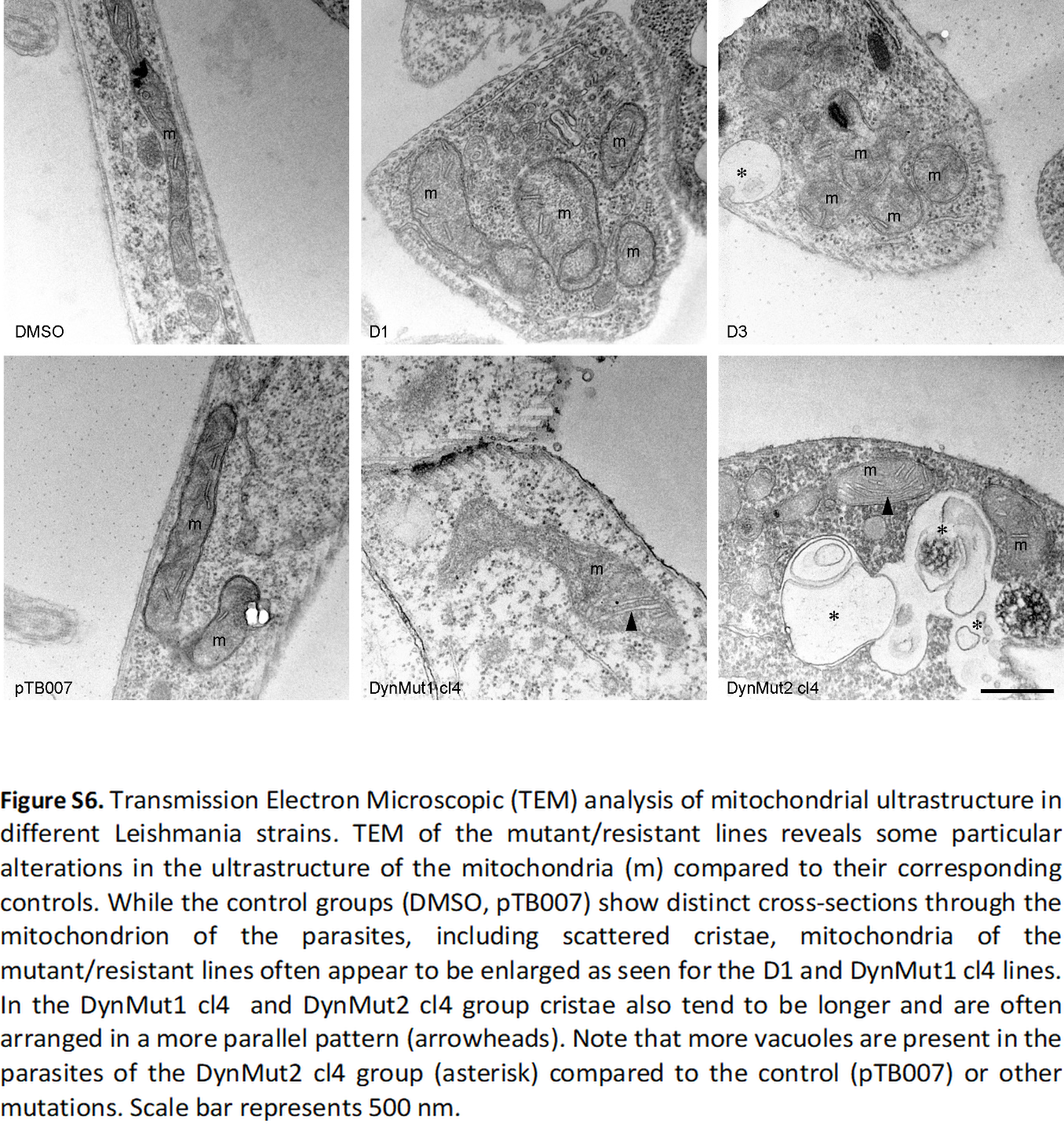

Supplement: FIG S6 [file mbio.03264-21-sf006.tif]

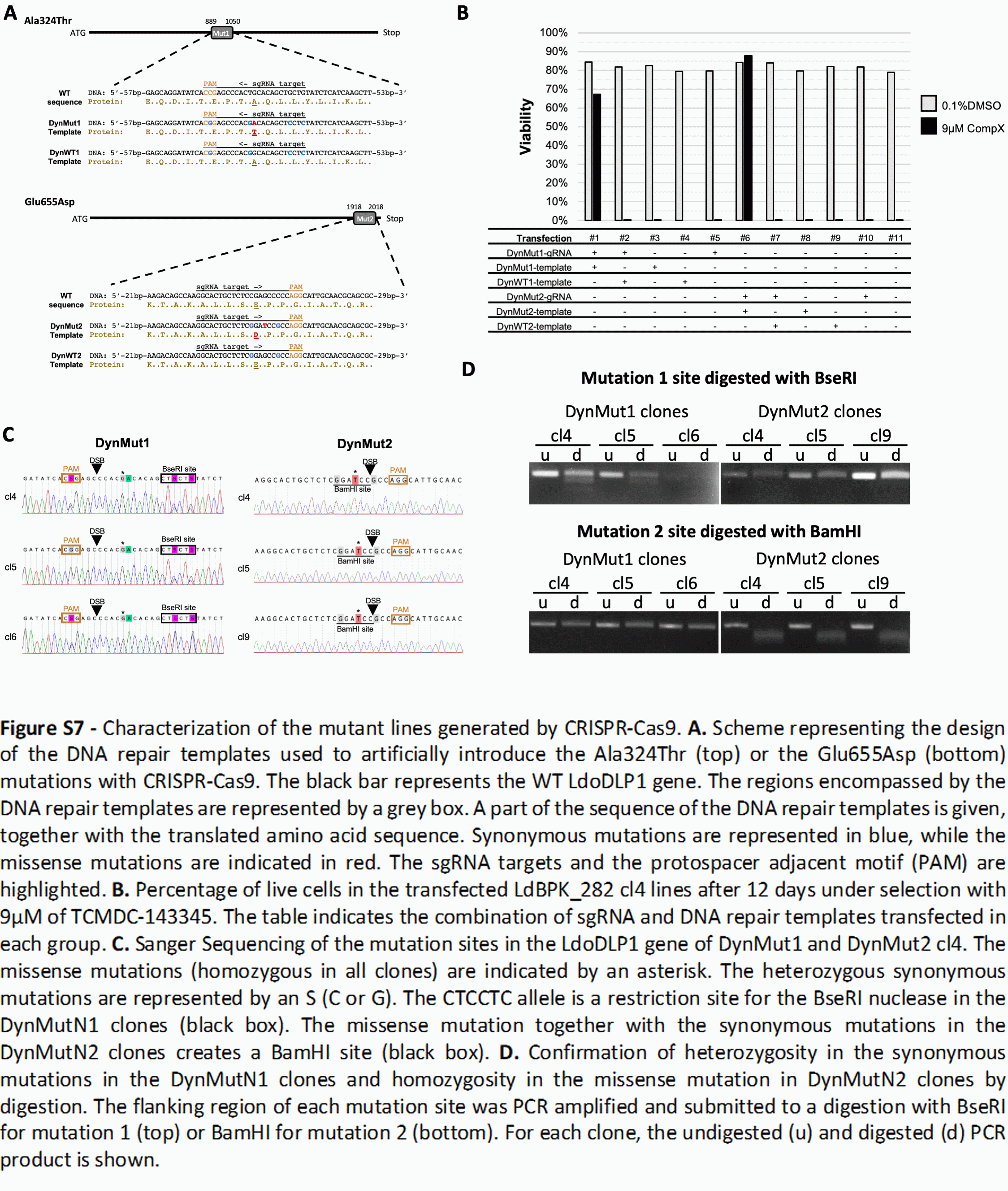

Supplement: FIG S7 [file mbio.03264-21-sf007.gif]
