# Supplementary material for: Genomic and Phenotypic Characterization of Experimentally Selected Resistant Leishmania donovani Reveals a Role for Dynamin-1-Like Protein in the Mechanism of Resistance to a Novel Antileishmanial Compound
Source: mBio. 2022 Jan 11;13(1):e03264-21. doi: 10.1128/mbio.03264-21 (PMC8749414; doi:10.1128/mbio.03264-21)
Supplement: TEXT S1 [file mbio.03264-21-s0001.docx]

**Supplementary text for the paper**

**Genetic and phenotypic characterization of experimentally selected resistant *Leishmania donovani* reveals a role for dynamin-1 like protein in the mechanism of resistance to a novel anti-leishmanial compound**

Aya Hefnawy^1^, Gabriel Negreira^1^, Marlene Jara^1^, James A. Cotton^2^, Ilse Maes^1^, Erika D’ Haenens^1^, Hideo Imamura^1^, Bart Cuypers^1^, Pieter Monsieurs^1^, Christina Mouchtoglou^1^, Hans De Winter^3^, Isabel Pintelon^4^, Jean-Pierre Timmermans^4^, Matt Berriman^2^, Mandy Sanders^2^, Julio Martin^5^, Geraldine de Muylder^1^, Jean-Claude Dujardin^1,6^, Yann G.-J. Sterckx^7*^, Malgorzata Anna Domagalska^1*^

^1^ Molecular Parasitology Unit, Institute of Tropical Medicine, Antwerp, Belgium

^2^ Wellcome Sanger Institute, Wellcome Genome Campus, Hinxton, UK

^3^ Laboratory of Medicinal Chemistry, University of Antwerp, Belgium

^4^  Department of Veterinary Sciences, University of Antwerp, Belgium

^5^ Global Health R&D, GlaxoSmithKline, Tres Cantos, Madrid, Spain

^6^ Department of Biomedical Sciences, University of Antwerp, Belgium

^7^ Laboratory of Medical Biochemistry and the Infla-Med Centre of Excellence, University of Antwerp, Belgium

* Corresponding authors

**Table of contents**

1. Supplementary Methods:

1.1. Promastigotes and susceptibility tests p.3

1.2. Mammalian cell cultures p.4

1.3. Multiple regression model for the evaluation of the effect of resistance

and engineered mutations on parasite growth p.4

1.4. Mapping of sequencing reads, SNP/indel prediction and gene ontology p.4

1.5. Somy of chromosomes p.5

1.6. CRISPR-Cas9 mediated engineering of *Leishmania* p.6

1.7. Phylogenetic analyses p.7

1.8. Transmission Electron Microscopy p.8

2. Results & Discussion

2.1. Growth curves of resistant and engineered lines p.8

2.2. Single nucleotide polymorphism p.9

2.3. Aneuploidy and local copy number variation p.10

2.4. CRISPR-Cas9-mediated introduction of point mutations in LdoDLP1 p.12

2.5. Homology modelling of LdoDLP1 p.13

3. References p.15

**Supplementary Methods**

***1.1. Promastigotes and susceptibility tests.***

We derived a line of LdBPK_282 cl4 from stabilate ITMAP031115A at passage number R36(13): meaning 36 passages since original isolation from patient, including 13 passages since cellular cloning (this was our Wild-Type line, WT). Promastigotes were maintained at 26 °C in hemoflagellate modified Eagles’s medium (HOMEM) supplemented with 20% (v/v) heat-inactivated FBS. TCMDC-143345 was purchased from Chemdiv. Another compound of the same family (currently called Compound Y, because of intellectual property sensitivity) was provided from DNDi, while the antileishmanial reference drugs Sb^III^ (potassium antimonyl tartrate trihydrate), MIL and AmphoB were purchased from Sigma-Aldrich.

Parasites were added to the wells of a 96 well tissue culture plate and grown in the presence of medium alone (controls) or medium containing various concentrations of the compound of choice, ranging from 50 µM to 0.75 µM. Plates were incubated for 72 hours at 26°C. Then, resazurin solution was added at a final concentration of 50-80 μM per well and the samples were incubated for a further 4 hours at 26°C. The absorbance of samples at 550–590 nm was determined using a spectrophotometer (PerkinElmer 2030). The effect of drug treatment on cell viability was quantified with the IC_50_: this was calculated with GraphPad Prism using a sigmoidal dose-response model with variable slope.

***1.2. Mammalian cell cultures***

THP-1 cells (human monocytic leukemia, ATCC-TIB-202) were maintained in RPMI media (Life Technologies) supplemented with 10% heat-inactivated fetal bovine serum (FBS) (Gibco) and 50 μM 2-mercaptoethanol at 37˚C in 5% CO^2^.

***1.3. Multiple regression model for the evaluation of the effect of resistance and engineered mutations on parasite growth***

The first three days of the growth curve from the different lines were selected to build a multiple regression model and the amount of parasites/ml were used as a measurement of the growth capability of each line under optimal conditions. The parasites/mL was transformed to a log2 scale to obtain a linear response as explained by two variables the number of days and the line as a categorical factor where the wildtype was used as reference. The adjusted R^2^ was used as an indicator of how well the model fitted the data, the coefficients were used to calculate the magnitude of the effect (positive or negative) and the p values for each variable and category were used as indicator of a significant effect on the amount of parasites/mL.

***1.4. Mapping of sequencing reads, SNP/indel prediction and gene ontology***

Reads were mapped to the reference *L. donovani* genome LdBPKv2 (1) using Smalt v0.7.4 (<http://www.sanger.ac.uk/science/tools/smalt-0>). The reference genome was indexed using a k-mer size 13 (*-k* option) and step-size of 2 (*-s* option). Smalt options for exhaustive searching for optimal alignments (*-x* option) and random mapping of multiple hit reads (*-r* option) were used. Picard v1.92 (<http://broadinstitute.github.io/picard/>) was used for merging and sorting bam files and marking duplicated reads. SNPs and small insertions and deletions (indels) were called using population-based Unified Genotyper method in Genome Analysis Toolkit v3.4 (GATK: <https://software.broadinstitute.org/gatk/>). Low quality SNPs were filtered using GATK Variant Filtration with QD < 2.0 || MQ < 40 || FS > 60.0 || ReadPosRankSum < -8.0.

Gene ontology and KEGG pathway analyses were carried out with the g:Profiler tool (available at https://biit.cs.ut.ee/gprofiler). As *Leishmania major* is currently the only supported *Leishmania* species for this tool, we orthology mapped the gene identifiers of BPK282v2 to the latest version of the *Leishmania major* genome, derived from TriTrypDB version 46. For orthology mapping, we used OrthoFinder v2.3.11 using default settings. When 1 *L. donovani* gene mapped to multiple (closely related) *L. major* genes, only one was randomly kept to prevent bias towards this gene. To avoid false positive results, we only considered GO enrichments to be significant if the following criteria were met: 1) FDR Adjusted p-value < 0.05, 2) at least five genes associated with the GO term in the enriched fraction, or three when small gene-sets (<50 genes) were evaluated.

***1.5. Somy of chromosomes***

Noteworthy, given the important mosaicism in *Leishmania* (2), chromosome or local copy numbers calculated here from bulk sequencing data are always average values for a given population of cells. To calculate the somy values, the raw average depth and raw standard deviation for each chromosome were calculated first. Then the median depth per chromosome excluding the depth outside of one standard deviation from its average depth was calculated. The median of all the chromosome median depths was used to normalise and obtain somy values (S values). The range of monosomy, disomy, trisomy, tetrasomy, and pentasomy was defined to be the full cell-normalized chromosome depth or somy, with S < 1.5, 1.5 ≤ S < 2.5, 2.5 ≤ S < 3.5, 3.5 ≤ S < 4.5, and 4.5 ≤ S < 5.5, respectively.

***1.6. CRISPR-Cas9 mediated engineering of Leishmania***

The approach consisted of transfecting a sgRNA template targeting the mutation locus and a synthetic double-stranded oligo DNA (dsODN) repair template bearing the Ala324Thr or the Glu655Asp point mutation into WT LdBPK_282 cl4 parasites constitutively expressing the Cas9 and the T7RNA polymerase genes (see main text). The DNA repair templates also include synonymous mutations to prevent Cas9 attack once they are integrated in the genome (Fig. S7A). These synonymous mutations also create restriction sites used to determine if mutations are homozygous or heterozygous and allow the distinction between naturally occurring and artificially introduced mutations. The use of a dsODN demonstrated a higher mutation efficiency when compared to single-stranded templates of both strands (data not shown).

The DNA repair templates bear the G to A (Ala324Thr) or the G to T (Glu655Asp) missense mutations and include synonymous mutations which were designed following *L. donovani* codon usage frequencies to prevent impact on expression caused by potential codon bias. For each template, another construct was created bearing the synonymous mutations but lacking the missense mutation to work as controls. The repair templates were produced as double stranded DNA molecules by annealing of equimolar ratios of the oligos DynMutN1Cas-F and DynMutN1Cas-R for the Ala324Thr mutation, and DynMutN2Cas-F with DynMutN2Cas-R for the Glu655Asp mutation. Each oligo pair was heated at 95˚C for 5 minutes and gradually cooled down at a rate of -1˚C per minute until reaching 25˚C using a thermocycler. The control repair templates DynWT1 and DynWT2 were generated by the same approach using the oligos DynWTN1Cas-F and DynWTN1Cas-R for the Ala324Thr mutation, and DynWTN2Cas-F and DynWTN2Cas-R for the Glu655Asp mutation.

The complete coding sequence of the LdoDLP1 gene of the 6 mutant clones generated by CRISPR-Cas9 was amplified by PCR with primers Dyn1-F and Dyn1-R and submitted to Sanger Sequencing with primers Dyn_seq1-f, Dyn_seq2_r, Dyn1-R, DynMutN1_control-R, and DynMutN2-Flank-F.

***1.7. Phylogenetic analyses***

The amino acid sequences of the following DRPs were used (the numbers between brackets represent the Uniprot IDs): *Arabidopsis thaliana* DLP Arc5 (Q84N64), *A. thaliana* DRP1A (P42697), *A. thaliana* DRP2A (Q9SE83), *A. thaliana* DRP2B (Q9LQ55), *A. thaliana* DRP3B (Q8LFT2), *Caenorhabditis elegans* DRP1 (G5EDY8), *C. elegans* DYN1 (Q9U9I9), *Chaetomium thermophilum* Mgm1 (G0SGC7), *Cyanidioschyzon merolae* DNM1 (Q84Y91), *Dictyostelium discoideum* DNMA (Q94464), *Drosophila melanogaster* DNM (P27619), *D. melanogaster* DRP1 (Q9VQE0), *D. melanogaster* Opa1 (Q95U20), *Aspergillus nidulans* VpsA (Q8X230), *E. coli* CrfC (P0DM85), *Giardia intestinalis* DLP (Q8T6L2), *Glycine max* DRP12A (Q39821), *Homo sapiens* DLP1 (O00429), *H. sapiens* Opa1 (O60313), *H. sapiens* MxA (P20591), *H. sapiens* DNM1 (Q05193), *L. donovani* DLP1 (E9BL28), *Rattus norvegicus* DNM1 (P21575), *Saccharomyces cerevisiae* DNM1 (P54861), *S. cerevisiae* Mgm1 (P32266), *S. cerevisiae* Vps1 (P21576), and *Trypanosoma brucei* DLP1 (Q8ITV0).

**1.8. Transmission Electron Microscopy**

For transmission electron microscopy, pellets of each *Leishmania* line (5.10^6^ parasites/pellet) were fixed in 5 ml eppendorf tubes with 2.5% glutaraldehyde in 0.1 M sodium cacodylate-buffer (pH 7.4). Post-fixation was performed for 2 hrs at room temperature in 1% OsO4 solution (veronal acetate-buffered at pH 7.4) followed by a 1 hr incubation in 1% tannic acid in veronal acetate buffer. After dehydration in a graded ethanol series (50% - 70% - 90% - 95% - 100% ethanol), pellets were embedded in EMbed 812 (Electron Microscopy Sciences, Hatfield, Pennsylvania). Ultrathin sections were stained with lead citrate and examined using a Tecnai G2 Spirit Bio Twin Microscope (FEI, Eindhoven, The Netherlands) at 120 kV.

**2. Results & Discussion**

***2.1. Growth curves of resistant and engineered lines***

The growth curves of both wildtype and resistant lines had a sigmoidal appearance with logarithmic phase and stationary phase. However, the mutant lines had a moderated but significant lower growth rate than the wildtype. This was evident after a multiple linear regression was modeled for the logarithmic phase of the growth curve (D0-D3). The number of parasites/ml (in log2 scale) was modeled to be explained by both the day number in culture and the line as a categorical variable using the wildtype as reference. The model had an adjusted R^2^ = 0.93 and an intercept= 19.51. As expected, the day number had a positive effect in the number of parasites/mL with a positive coefficient equal to 1.67 while the coefficients for the Line C and Line D3 were -1.14 (p < 1.06e-05) and -0.51 (p< 0.026) respectively indicating their diminished rate of growth.

The effect of the LdoDLP1 engineered mutations on the parasite growth was modeled again with multiple regression during the logarithmic phase of the growth curve. The model had an adjusted R^2^ = 0.96 and an intercept= 19.09.  The day number had a positive effect in the number of parasites/mL with a coefficient equal to 1.77 while the coefficients for the DynMut1 and DynMut2 lines were -0.22 (p = 0.18) and -0.36 (p= 0.03) respectively. The coefficients indicate that although both DynMut1 and DynMut2 had a diminished growth rate in comparison to the wildtype the negative effect is only significant for DynMut2. However, no difference was observed when comparing both DynMut1 and DynMut2 and pT007 control, the WT line transfected with the CRISPR-Cas9.

***2.2. Single nucleotide polymorphisms***

Besides fixed missense mutations mentioned in the main text, other SNPs were observed in the selected resistant lines (extracted from DataS1B, only for annotated genes):

1. absent in C but heterozygous in D lines: upstream gene LdBPK_320013600 (CYC2-likecyclin, putative), downstream gene LdBPK_190009300 (protein kinase, putative) and upstream gene LdBPK_250016900 (MGT2 Magnesium transporter)
2. absent in D but heterozygous in C: upstream gene LdBPK_190019900 (membrane-bound O-acyltransferase,putative), upstream gene LdBPK_360031900 (zinc finger containing protein,putative), upstream gene LdBPK_340013800 (serine/threonine protein phosphatase PP1,putative)
3. absent in D but fixed in C: synonymous mutation in gene LdBPK_360080200 (adaptin complex 1subunit,putative)

An alternative way to identify the most significant SNPs with regards to the IC_50_ shift was to use a testing approach inspired by burden tests for rare disease associations (Fig. S3 D-G), reasoning that different variants in the same gene could contribute to a resistance phenotype (3). For each sample, we summed the frequency of all non-reference alleles at every variant within the coding sequence for each gene for each experimental sample and regressed this total burden of non-reference mutations against the IC_50_ for that sample. The LdoDLP1 gene showed the highest significance and a correlation R^2^ value of 0.63 (p value = 7x10^-9^) between total allele frequency and the IC_50_. Other significant genes had lower R^2^ values: 0.44 (protein kinase, p value = 6x10^-6^), 0.48 (MgT2 transporter, p value = 1.6x10^-6^) and 0.41 (mitochondrial RNA binding complex 1 subunit, p value = 1.5x10^-5^).

***2.3. Aneuploidy and local copy number variation***

No somy changes were observed in the WT or the DMSO lines along the selection and patterns of lines D1-4 were very similar (Fig. S3A); we thus focused on a deeper analysis of lines C and D1 (Fig. 2). More somy changes were observed in Line C than Line D1 with significant changes observed in 12 chromosomes vs. 6 chromosomes, respectively. There were three somy changes common to both lines: chr6 (disomy to trisomy from 4 μM in line C and 6 μM in line D1), chr31 (tetrasomy to pentasomy from 4 μM in line C and 6 μM in line D1) and chr35 (disomy to trisomy at 5 and 8 μM in line C and starting from 6 μM in line D1). In line D1 change from disomy to trisomy appeared at 6μM for chr3 and 12. A GO and Kegg Pathway enrichment analysis was performed on the complete gene content of chr6 and chr31, respectively (Data S1C). No GO classes nor Kegg Pathways were significantly enriched on chromosome 6. In contrast, for chromosome 31 all three GO classes showed significant enrichment. This included: amino acid transmembrane transport (Biological Process), transmembrane transport activity and calcium-dependent cysteine-type endopeptidase activity (Molecular Function) and integral component of membrane (Cellular Compartment) (not exhaustive, full list in Data S1C). With regards to KEGG pathways, there was a significant enrichment for genes involved in valine, leucine, and isoleucine degradation.

Secondly the local CNVs have been investigated. We found only one significant CNV (independently of somy change) affecting a single gene: a duplication of Elongation factor1-alpha (chr17) was observed in line D1 at 4 μM of selection pressure yet this increase of copy number was not stable, as it disappeared afterwards in the selection process. Variation in copy number of long chromosomal stretches were observed: (i) an increase in the copy number (from 2 to 4) of a sub-telomeric segment of 56 genes of chr17 in line D1 occurred at 4 μM selection pressure, but copy number went back to 2 at higher drug concentration and (ii) a partial loss of 42 genes in a subtelomeric fragment of chr30 was observed in line C at highest drug concentration (12 μM; Fig. S3 B-C). The list of affected genes is shown in Data S1C. With regards to GO enrichments, none were found for the fragment of Chr30, but several molecular functions were enriched for the fragment of chr17 predominantly related to fatty-acyl-CoA binding (Data S1C).

Whole genome sequencing is a powerful tool to identify molecular changes accompanying the development of drug resistance (DR) (4–6). Differential expression of specific genes through extra- or intra-chromosomal amplicons or through aneuploidy is a commonly reported mechanism during experimental selection of DR in *Leishmania* (7). Indeed, in the absence of gene-specific transcription factors (8), localized gene amplifications/deletions and somy changes constitute a rapid way for the parasite to modulate gene dosage and adapt to selective environment like drug pressure (1). We observed here large subtelomeric amplifications/deletions at a single time point in the TCMDC-143345-resistance selection process, but they disappeared later and thus probably did not play a major role in the final phenotype of resistance to TCMDC-143345. In contrast, several aneuploidy changes were observed (involving 5 to 12 chromosomes, mostly amplifications) and some of them remained fixed until highest resistance. From our work, it is not possible to discern if the selection is driven by the entire amplicons/chromosomes or only by one or several genes present in these.

***2.4.* CRISPR-*Cas9-mediated introduction of mutations in LdoDLP1***

In the populations transfected with DynMut1-gRNA and DynMut1-template (Ala324Thr), or DynMut2-gRNA and DynMut2-template (Glu655Asp) and submitted to selection with 9 µM TCMDC-143345 24h after transfection, a high percentage of viable cells was observed after 12 days under continuous drug pressure (Fig. S7B, transfections #1 and #6 respectively). When the donor DNA was replaced by a dsODN lacking the missense mutation (transfections #2 and #7), or when sgRNA templates were omitted (transfections #3, #4, #8 and #9), no parasite survived the selection. Taken together, these results reveal that the introduction of either the Ala324Thr or the Glu655Asp mutations in a WT background conferred to the promastigotes the ability to survive and grow under the presence of TCMDC-143345.

Next, 3 clones of each selected line were isolated and the complete LdoDLP1 gene was sequenced. The sequencing confirmed the presence of the missense and synonymous mutations in all clones (Fig. S7C). The absence of overlapping peaks in the missense mutations in all clones indicate that these mutations were homozygous. However, in the DynMut1 clones, some synonymous mutations (3 in clones 4 and 6 and 2 in clone 5) were present as heterozygous. Since the G to C synonymous mutations create a BseRI restriction site, heterozygosity was confirmed by the observation that this region is only partially digested by this enzyme (Fig. S7D - top). For the DynMut2, the introduced point mutations create a BamHI site. The complete digestion of the region encompassing these mutations with this enzyme in DynMutN2 clones confirm homozygosity (Fig. S7D – bottom). No other mutation was present in the LdoDLP1 gene in any clone.

***2.5. Homology modelling of LdoDLP1***

Homology modeling reveals that LdoDLP1 possesses all the structural features necessary to execute mitochondrial fission according to the “hydrolysis-dependent powerstroke” model underlying DRP function (Fig. 6 and (9)). Briefly, this model proposes that the following steps lead to fission of the outer mitochondrial membrane: i) LdoDLP1 dimers bind to the membrane region to be constricted through their foot domains, ii) membrane-bound LdoDLP1 dimers cooperatively assemble into higher-order oligomers through a specific oligomerisation interface (Fig. 6 C, box ‘1’), iii) GTP-binding induces dimerization of the GTPase domains between tetramers in adjacent helical rungs for optimal positioning during LdoDLP1’s “hydrolysis-dependent powerstroke”, iv) GTP hydrolysis leads to a conformational change in LdoDLP1 that relies on flexibility conferred by the ‘Hinge 1’ region (Fig. 6C, box ‘2’), v) this “hydrolysis-dependent powerstroke” propagates through the LdoDLP1 helical rungs which leads to further constriction of the encompassed membrane area and eventually results in membrane fission. Interestingly, the LdoDLP1 Ala324Thr and Glu655Asp mutations associated with TCMDC-143345 resistance are located in the protein’s oligmerisation interface and ‘Hinge 1’ region, respectively, two regions that are essential for DRP (and thus LdoDLP1) function.

The Ala324Thr mutation maps onto a part of the stalk domain that is closely located to the α2-helix of the neck domain (Fig. 6B). Structural work performed for other DRPs has identified this region as the interface responsible for the higher-order oligomerization of DRP dimers(10) (11) (12) (13). In the case of LdoDLP1, this interface is mainly formed through hydrophobic interactions, which is in accordance with the available DRP models (Fig. 6C, box ‘1’). Ala324 is positioned on the outer rim of the oligomerization interface and its substitution by a slightly bulkier amino acid such as Thr could affect the properties of this interface. Hence, an Ala324Thr mutation could alter the tendency of LdoDLP1 dimers to form higher-order oligomers, which could in turn influence protein function.

The Glu655Asp mutation is located in a region known as ‘Hinge 1’(13) (14), which connects the stalk domain with the α3-helix of the neck domain (Fig. 6B). ‘Hinge 1’ confers flexibility to DRPs, which is important for the so-called “hydrolysis-dependent powerstroke” underlying protein function(9). This flexibility originates from relative movements between i) the neck and stalk domains and ii) the GTPase and neck domains. Relative domain movements between the neck and stalk are limited due to an extensive hydrophobic interaction network(14), which is also found in LdoDLP1 (Fig. 6C, box ‘2’). Hence, the bulk of the flexibility is provided by relative motions between the GTPase and neck domains and is mediated by a salt bridge between an Arg - Glu pair that tends to be well conserved within DRP members. In the case of *H. sapiens* MxA it has been demonstrated that mutation of the conserved Arg (Arg640) to an Ala abolishes ‘Hinge 1’ flexibility(14). For LdoDLP1, this conserved residue pair corresponds to Arg663 - Glu655 (Fig. 6C, box ‘2’). Replacing a Glu with an Asp at position 655 would significantly increase the distance between the interacting side chains of the Arg663 - Asp655 pair and would thus be expected to weaken the salt bridge. Hence, a Glu655Asp mutation could have a considerable impact on the flexibility of LdoDLP1’s ‘Hinge 1’ region and, thus, protein function.

1. **References**

1. Dumetz F, Imamura H, Sanders M, Seblova V, Myskova J, Pescher P, Vanaerschot M, Meehan CJ, Cuypers B, De Muylder G, Späth GF, Bussotti G, Vermeesch JR, Berriman M, Cotton JA, Volf P, Dujardin JC, Domagalska MA. 2017. Modulation of aneuploidy in *Leishmania donovani* during adaptation to different in vitro and in vivo environments and its impact on gene expression. MBio 8:e00599-17.

2. Sterkers Y, Lachaud L, Crobu L, Bastien P, Pagès M. 2011. FISH analysis reveals aneuploidy and continual generation of chromosomal mosaicism in *Leishmania major*. Cell Microbiol 13:274–83.

3. Lee S, Abecasis GR, Boehnke M, Lin X. 2014. Rare-variant association analysis: Study designs and statistical tests. Am J Hum Genet. 95: 5-23

4. Dumetz F, Cuypers B, Imamura H, Zander D, D’Haenens E, Maes I, Domagalska MA, Clos J, Dujardin J-C, De Muylder G. 2018. Molecular Preadaptation to Antimony Resistance in *Leishmania donovani* on the Indian Subcontinent . mSphere 3:e00548-17.

5. Shaw CD, Imamura H, Downing T, Blackburn G, Westrop GD, Cotton JA, Berriman M, Sanders M, Rijal S, Coombs GH, Dujardin JC, Carter KC. 2019. Genomic and Metabolomic Polymorphism among Experimentally Selected Paromomycin-Resistant *Leishmania donovani* Strains. Antimicrob Agents Chemother 64:e00904-19.

6. Shaw CD, Lonchamp J, Downing T, Imamura H, Freeman TM, Cotton JA, Sanders M, Blackburn G, Dujardin JC, Rijal S, Khanal B, Illingworth CJR, Coombs GH, Carter KC. 2016. In vitro selection of miltefosine resistance in promastigotes of *Leishmania donovani* from Nepal: Genomic and metabolomic characterization. Mol Microbiol 99:1134–1148.

7. Leprohon P, Fernandez-Prada C, Gazanion É, Monte-Neto R, Ouellette M. 2015. Drug resistance analysis by next generation sequencing in *Leishmania*. Int J Parasitol Drugs Drug Resist. 5(1):26-35

8. De Gaudenzi JG, Noé G, Campo VA, Frasch AC, Cassola A. 2011. Gene expression regulation in trypanosomatids. Essays Biochem 51:31–46.

9. Chappie JS, Mears JA, Fang S, Leonard M, Schmid SL, Milligan RA, Hinshaw JE, Dyda F. 2011. A pseudoatomic model of the dynamin polymer identifies a hydrolysis-dependent powerstroke. Cell 147:209–222.

10. Kalia R, Wang RYR, Yusuf A, Thomas P V., Agard DA, Shaw JM, Frost A. 2018. Structural basis of mitochondrial receptor binding and constriction by DRP1. Nature 558:401–405.

11. Faelber K, Dietrich L, Noel JK, Wollweber F, Pfitzner AK, Mühleip A, Sánchez R, Kudryashev M, Chiaruttini N, Lilie H, Schlegel J, Rosenbaum E, Hessenberger M, Matthaeus C, Kunz S, von der Malsburg A, Noé F, Roux A, van der Laan M, Kühlbrandt W, Daumke O. 2019. Structure and assembly of the mitochondrial membrane remodelling GTPase Mgm1. Nature. 571: 429-433.

12. Reubold TF, Faelber K, Plattner N, Posor Y, Ketel K, Curth U, Schlegel J, Anand R, Manstein DJ, Noé F, Haucke V, Daumke O, Eschenburg S. 2015. Crystal structure of the dynamin tetramer. Nature 525:404–408.

13. Gao S, Von der Malsburg A, Dick A, Faelber K, Schröder GF, Haller O, Kochs G, Daumke O. 2011. Structure of Myxovirus Resistance Protein A Reveals Intra- and Intermolecular Domain Interactions Required for the Antiviral Function. Immunity 35:514–525.

14. Chen Y, Zhang L, Graf L, Yu B, Liu Y, Kochs G, Zhao Y, Gao S. 2017. Conformational dynamics of dynamin-like MxA revealed by single-molecule FRET. Nat Commun 8:15744.
