# Supplementary material for: Genomic and Phenotypic Characterization of Experimentally Selected Resistant Leishmania donovani Reveals a Role for Dynamin-1-Like Protein in the Mechanism of Resistance to a Novel Antileishmanial Compound
Source: mBio. 2022 Jan 11;13(1):e03264-21. doi: 10.1128/mbio.03264-21 (PMC8749414; doi:10.1128/mbio.03264-21)
Supplement: TABLE S1 [file mbio.03264-21-st001.docx]

**Table S1** - List of primers and oligos used for CRISPR-Cas9 mediated engineering

| **Primers used in the Cloning of the LdoDLP1 gene into the pLEXSy_Hyg vector** | | |
| --- | --- | --- |
| **Primer Name** | **Primer Sequence** | **OBS:** |
| InF-LdDNM1-F | ACCAGATCTGCCATGGACCAGTTGATCAGCGTGATC |  |
| InF-LdDNM1-R | AGGAGGAGGGCGGCCGCTTAGGCGCCGGCTTGCATGG |  |
| **Primers used to amplify the complete CDS of LdoDLP1 gene for sanger sequencing** | | |
| Dyn1-F | ATGGACCAGTTGATCAGCGTGATC |  |
| Dyn1-R | TTAGGCGCCGGCTTGCATG |  |
| **Primers used in the Sanger Sequencing of the LdoDLP1 gene from gDNA** | | |
| Dyn_seq1_f | CAACACCGATCTTGCCACGA |  |
| Dyn_seq2_r | CCAGCGCAGATTTCGACGAT |  |
| Dyn1-R | TTAGGCGCCGGCTTGCATG |  |
| DynMutN1_control-R | GTCGCTGAAAAGCTTGATGAG |  |
| DynMutN2-Flank-F | GACGATGCACGAGCAGTACATG |  |
| **Primers used to generate the gRNA template for to introduce the Ala324Thr mutation** | | |
| G00 | AAAAGCACCGACTCGGTGCCACTTTTTCAAGTTGATAACGGACTAGCCTTATTTTAACTTgctatttctagctctaaaac | Lowercase: annealing site to gRNA primer. |
| DynMutN1-gRNA | gaaattaatacgactcactataggCAGCAGCTGTGCAGTGGGCTgttttagagctagaaatagc | Underlined: T7 promoter. Uppercase: gRNA target |

| **Primers used to generate the gRNA template for to introduce the Glu655Asp mutation** | | |
| --- | --- | --- |
| G00 | AAAAGCACCGACTCGGTGCCACTTTTTCAAGTTGATAACGGACTAGCCTTATTTTAACTTgctatttctagctctaaaac | Lowercase: annealing site to gRNA primer. |
| DynMutN2-gRNA | gaaattaatacgactcactataGGCACTGCTCTCCGAGCCCCCgttttagagctagaaatagc | Underlined: T7 promoter. Uppercase: gRNA target |
| **Oligos used to generate the donor DNA molecules for the Ala324Thr mutation** | | |
| DynMutN1Cas-F | CACGTGGACCAGCTGATGGAAGCCACCAAGAAGCAGATGGAGAAGCTGGGCATGTTTGAGCAGGATATCAC**G**GAGCCCAC**GA**CACAGCT**C**CT**C**TATCTCATCAAGCTTTTCAGCGACACGCTGAATCAGACGATTGATGGTGGCATCACGGATGCCACGAAG | Bold: Synonymous mutations;  Red: missense mutation. |
| DynWTN1Cas-F | CACGTGGACCAGCTGATGGAAGCCACCAAGAAGCAGATGGAGAAGCTGGGCATGTTTGAGCAGGATATCAC**G**GAGCCCAC**G**GCACAGCT**C**CT**C**TATCTCATCAAGCTTTTCAGCGACACGCTGAATCAGACGATTGATGGTGGCATCACGGATGCCACGAAG | Bold: Synonymous mutations; |
| DynMutN1Cas-R | CTTCGTGGCATCCGTGATGCCACCATCAATCGTCTGATTCAGCGTGTCGCTGAAAAGCTTGATGAGATA**G**AG**G**AGCTGTG**TC**GTGGGCTC**C**GTGATATCCTGCTCAAACATGCCCAGCTTCTCCATCTGCTTCTTGGTGGCTTCCATCAGCTGGTCCACGTG | Bold: Synonymous mutations;  Red: missense mutation. |
| DynWTN1Cas-R | CTTCGTGGCATCCGTGATGCCACCATCAATCGTCTGATTCAGCGTGTCGCTGAAAAGCTTGATGAGATA**G**AG**G**AGCTGTGC**C**GTGGGCTC**C**GTGATATCCTGCTCAAACATGCCCAGCTTCTCCATCTGCTTCTTGGTGGCTTCCATCAGCTGGTCCACGTG | Bold: Synonymous mutations; |
| **Oligos used to generate the donor DNA molecules for the Glu655Asp mutation** | | |
| DynMut-N2Cas-F | GTGAGGGCCTCGAGCATCTCTTTGGCCGCCTTGCGCTGCGTTGCAATGCCTGG**C**GG**A**TC**C**GAGAGCAGTGCCTTGGCTGTCTTGTCCGAGTACAGCTCACTCACCAGGCGCGCATAGACTT | Bold: Synonymous mutations;  Red: missense mutation. |
| DynWT-N2Cas-F | GTGAGGGCCTCGAGCATCTCTTTGGCCGCCTTGCGCTGCGTTGCAATGCCTGG**C**GGCTC**C**GAGAGCAGTGCCTTGGCTGTCTTGTCCGAGTACAGCTCACTCACCAGGCGCGCATAGACTT | Bold: Synonymous mutations; |
| DynMut-N2Cas-R | AAGTCTATGCGCGCCTGGTGAGTGAGCTGTACTCGGACAAGACAGCCAAGGCACTGCTCTC**G**GA**T**CC**G**CCAGGCATTGCAACGCAGCGCAAGGCGGCCAAAGAGATGCTCGAGGCCCTCAC | Bold: Synonymous mutations;  Red: missense mutation. |
| DynWT-N2Cas-R | AAGTCTATGCGCGCCTGGTGAGTGAGCTGTACTCGGACAAGACAGCCAAGGCACTGCTCTC**G**GAGCC**G**CCAGGCATTGCAACGCAGCGCAAGGCGGCCAAAGAGATGCTCGAGGCCCTCAC | Bold: Synonymous mutations; |
